# Inhibition of PKC-δ retards kidney fibrosis via inhibiting cGAS-STING signaling pathway in mice

**DOI:** 10.1038/s41420-024-02087-z

**Published:** 2024-07-07

**Authors:** Dongyun Wang, Yue Li, Guiying Li, Mengyu Liu, Zihui Zhou, Ming Wu, Shan Song, Yawei Bian, Jiajia Dong, Xinran Li, Yunxia Du, Tao Zhang, Yonghong Shi

**Affiliations:** 1 Department of Pathology, Hebei Medical University, Shijiazhuang, 050017 China; 2 Hebei Key Laboratory of Kidney Disease, Shijiazhuang, 050017 China; 3 Department of Nephrology, Affiliated Hospital of Hebei University of Engineering, Handan, 056000 China; 4 Department of Nephrology, Third Hospital of Hebei Medical University, Shijiazhuang, 050051 China

**Keywords:** Kidney diseases, Chronic inflammation

## Abstract

Kidney fibrosis is considered to be the ultimate aggregation pathway of chronic kidney disease (CKD), but its underlying mechanism remains elusive. Protein kinase C-delta (PKC-δ) plays critical roles in the control of growth, differentiation, and apoptosis. In this study, we found that PKC-δ was highly upregulated in human biopsy samples and mouse kidneys with fibrosis. Rottlerin, a PKC-δ inhibitor, alleviated unilateral ureteral ligation (UUO)-induced kidney fibrosis, inflammation, VDAC1 expression, and cGAS-STING signaling pathway activation. Adeno-associated virus 9 (AAV9)-mediated VDAC1 silencing or VBIT-12, a VDAC1 inhibitor, attenuated renal injury, inflammation, and activation of cGAS-STING signaling pathway in UUO mouse model. Genetic and pharmacologic inhibition of STING relieved renal fibrosis and inflammation in UUO mice. In vitro, hypoxia resulted in PKC-δ phosphorylation, VDAC1 oligomerization, and activation of cGAS-STING signaling pathway in HK-2 cells. Inhibition of PKC-δ, VDAC1 or STING alleviated hypoxia-induced fibrotic and inflammatory responses in HK-2 cells, respectively. Mechanistically, PKC-δ activation induced mitochondrial membrane VDAC1 oligomerization via direct binding VDAC1, followed by the mitochondrial DNA (mtDNA) release into the cytoplasm, and subsequent activated cGAS-STING signaling pathway, which contributed to the inflammation leading to fibrosis. In conclusion, this study has indicated for the first time that PKC-δ is an important regulator in kidney fibrosis by promoting cGAS-STING signaling pathway which mediated by VDAC1. PKC-δ may be useful for treating renal fibrosis and subsequent CKD.

## Introduction

Chronic kidney disease (CKD) remains a worldwide public health concern affecting 8–15% of the global population [1]. Renal tubulointerstitial fibrosis is widely thought to be the common pathway that leads to end-stage renal failure in almost all progressive CKD, and it is also a crucial pathological manifestation of end-stage renal disease [2]. Kidney fibrosis is characterized by excessive deposition of extracellular matrix proteins such as fibronectin and collagens, and increased generation of α-smooth muscle actin (α-SMA) in the interstitium [3]. Collecting evidence demonstrated that the infiltration of inflammatory cells, and accompanied by increased expression of monocyte chemoattractant protein-1(MCP-1), interleukin 1β (IL-1β), tumor necrosis factor α (TNF-α), and interleukin 6 (IL-6) are involved in the renal fibrosis process [4]. However, the underlying mechanism of tubulointerstitial fibrosis remain obscure, and the treatment is lacking.

Protein Kinase C (PKC) is a family of serine/threonine kinases with several subtypes, including traditional PKCs (α, β, γ), novel PKCs (δ, ε, θ, η), and atypical PKCs (ζ, λ/ɩ) [5, 6]. PKC-δ also called as PRKCD, which has the ability to regulate cell growth, differentiation, apoptosis, transformation and tumorigenicity [7]. Previous study showed that cardiac ischemia and reperfusion led to the translocation of PKC-δ to mitochondria then affected the activity of downstream apoptotic factors through the release of cytochrome C [8]. PRKCD is localized to mitochondria and regulates recruitment of ULK1-ATG13 upon induction of mitophagy [9, 10]. In addition, PKC-δ inhibition protected against cisplatin nephrotoxicity by upregulating autophagy [11]. Meanwhile, inhibition of PKC-δ attenuated glycerine-induced renal damage in mice, and PKC-δ mediated myoglobin-induced apoptosis and expression of TNF-α and IL-1β in renal tubular cells [12]. Blockade of PKC-δ inhibited albumin-induced apoptosis in rat kidney proximal tubular cell line, and albumin overload-induced apoptosis in renal tubule in mice [13]. However, the role of PKC-δ in renal fibrosis has not been reported.

Various studies have revealed that mitochondrial dysfunction is associated with the pathogenesis of kidney fibrosis [14–16]. Renal tubular epithelial cells have abundant mitochondria, which require high energy to meet the huge energy demand of tubule absorption and secretion, and are particularly sensitive to various injuries such as hypoxia, oxidative stress and toxins [17]. The cyclic guanosine monophosphate-AMP synthase (cGAS) is a nucleic acid receptor located in the cytoplasm that recognizes double-stranded DNA from various sources present in the cytoplasm and combines with it to form dimers, then undergo conformational changes of cGAS to initiate immune response through activation of interferon gene stimulating factor (STING) pathway [18]. STING, also known as TMEM173, MITA, ERIS and MPYS, is an endoplasmic reticulum (ER) dimer linker protein and regarded as a central signaling component of intracellular DNA sensing pathways [19, 20]. It was verified that cytoplasmic mtDNA which released from mitochondria is a very important trigger for cGAS-STING activation [21, 22]. Previous studies have demonstrated that renal tubular cells mitochondrial damage caused inflammation via activating cGAS-STING signaling in acute kidney injury [23, 24]. In addition, cGAS-STING activation in glomeruli contributed to the podocyte injury in diabetic kidney disease [25, 26]. A recent study showed that genetic deletion of STING and pharmacologic STING inhibitor ameliorated folic acid-induced kidney fibrosis and inflammation in mice [27]. This finding strongly suggest that inhibition of cGAS-STING signaling activation may be a new therapeutic targets for renal fibrosis, which requires extensive research to confirm. Until now, the role of cGAS-STING signaling pathway in UUO-induced kidney injury has not been reported.

The voltage-dependent anion channel 1 (VDAC1) is a multi-functional protein, expressed in the mitochondria and other cell compartments, including the plasma membrane. Accumulating data indicate that VADC1 is involved in multiple cellular biological processes, such as oxidative stress, apoptosis, and metabolism [28]. Previous study has shown that the increase of VDAC oligomer pores promoted mitochondrial outer membrane permeability, and release of mitochondrial DNA (mtDNA) into the cytoplasms, leading to the activation of cGAS-STING signal and triggering inflammation [29]. In addition, mtDNA interact with the positively charged residues in the N-terminal domain of VDAC1 and facilitates oligomerization of VDAC1 [30]. VDAC1 oligomerization has been implicated in cisplatin-induced apoptosis due to pores formation in the outer mitochondrial membrane, which lead to release of cytochrome C from mitochondria to cytosol in renal cells [31]. Deletion of VDAC1 abrogated recovery of mitochondrial function, renal morphology and kidney function, and exacerbated kidney fibrosis in ischemia/reperfusion (I/R)-induced acute kidney injury [32]. In contrast, proximal tubule-specific VDAC1 knockout ameliorated I/R-induced renal injury, tubular cell apoptosis, and mitochondrial damage in mice [33]. However, whether it plays a role in renal fibrosis and underling mechanism remain unknown.

In the present study, we established a unilateral ureteral obstruction model in STING knockout mice. Meanwhile, UUO mice were treated with PKC-δ inhibitor Rottlerin, AAV-shVDAC1, VDAC1 inhibitor VBIT-12, and STING inhibitor C-176. We found that inhibition of PKC-δ, VDAC1 or STING alleviated kidney inflammation and fibrosis in UUO mice. In addition, hypoxia-induced inflammatory and fibrotic responses were abolished by inhibition of PKC-δ, VDAC1 or STING in HK-2 cells. PKC-δ activation induced mitochondrial membrane VDAC1 oligomerization, subsequentially caused release of mtDNA into the cytoplasm, and activation of cGAS-STING signaling pathway, which triggered renal inflammation and fibrosis.

## Results

### PKC-δ expression is upregulated in fibrotic kidneys

**Fig. 1:**
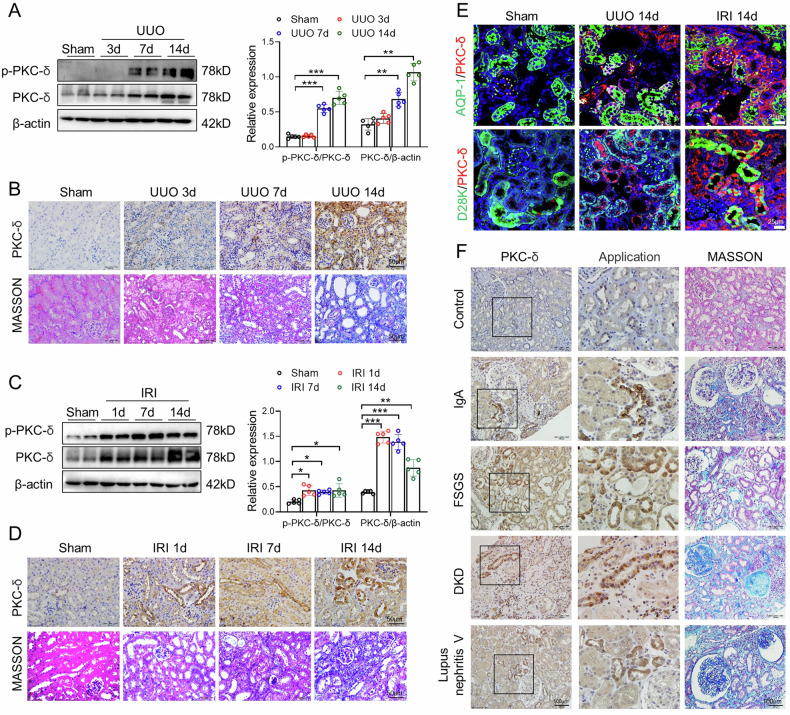
PKC-δ expression is upregulated in fibrotic kidneys. **A** Representative PKC-δ and p-PKC-δ western blot images and quantitation in kidneys at 3, 7 and 14 days after UUO (n = 5). **B** Representative images of immunochemistry staining of PKC-δ and Masson staining in kidneys of sham and UUO model. Bar = 50 μm. **C** Representative PKC-δ and p-PKC-δ western blot images and quantitation in kidneys at day 1, 7 and 14 after ischemia/reperfusion injury (IRI) (n = 5). **D** Representative images of immunochemistry staining of PKC-δ and Masson staining in sham and I/R-treated mouse kidneys. Bar = 50 μm. **E** Representative images of co-expression of PKC-δ and AQP-1, and co-expression of PKC-δ and calbindin-D28k in kidneys of Sham, UUO and IRI mice. Bar = 25 μm. **F** Representative images of immunochemistry staining of PKC-δ and Masson staining in Control, IgA, FSGS, DKD and Lupus nephritis V human kidney specimens. Bar = 100 μm. *P < 0.05, **P < 0.01, ***P < 0.001.

As shown in Fig. 1A, p-PKC-δ and PKC-δ expressions were increased in kidneys after UUO 7 and 14 days in mice. Immunohistochemical staining showed that PKC-δ expression was enhanced along with the collagen deposition in UUO kidneys (Fig. 1B). We also found that the expression levels of p-PKC-δ and PKC-δ were upregulated in IRI kidneys at day 1, 7 and 14 (Fig. 1C). In addition, the expression of PKC-δ was accompanied by the exacerbation of renal fibrosis (Fig. 1D). To confirm the localization of PKC-δ in vivo, we performed dual immunofluorescence staining of PKC-δ and tubular markers in UUO and IRI kidneys. PKC-δ was highly expressed in AQP-1 positive proximal tubules and D28K positive distal tubules after UUO or IRI (Fig. 1E). Next, we used immunohistochemical staining to investigate PKC-δ expression in kidney biopsies from patients with CKD, including IgA nephropathy, focal segmental glomerulosclerosis (FSGS), diabetic kidney disease (DKD), and class V Lupus nephritis (Fig. 1F). The results showed that PKC-δ expression was upregulated in renal tubular cells of renal biopsy specimens with CKD compared with control kidneys.

### Inhibition of PKC-δ ameliorates kidney fibrosis, inflammation, VDAC1 expression and activation of cGAS-STING pathway in UUO mice

**Fig. 2:**
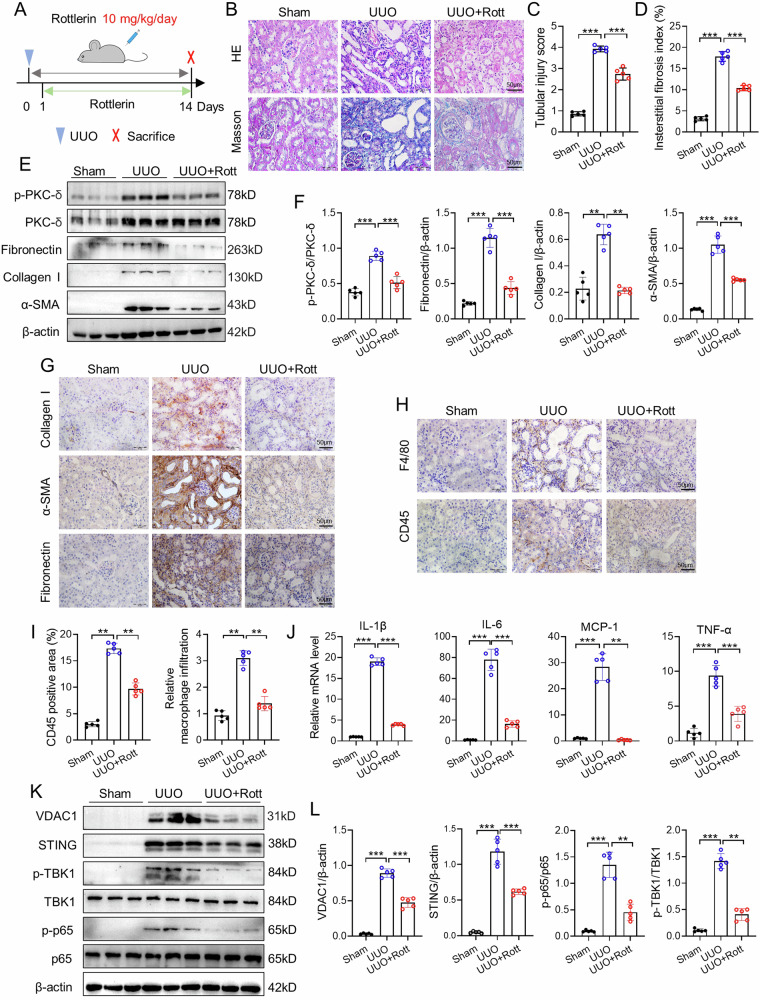
Inhibition of PKC-δ activation ameliorates kidney fibrosis, inflammation, VDAC1 expression and cGAS-STING pathway in UUO mice. The mice were randomly separated into the Sham, UUO and UUO+Rottlerin groups. **A** Schematic diagram of the experimental design: wild-type C57BL/6 mice were performed UUO surgery and treated with PKC-δ inhibitor (Rottlerin) for 13 days. The gray left and right double arrows indicate 14 days after UUO surgery, the green arrowheads indicate the injections of Rottlerin (10 mg/kg body weight). **B**–**D** Representative images of H&E staining and Masson staining in kidneys of the three groups, and corresponding quantitative analyses (n = 5). Bar = 50 μm. **E**, **F** Representative PKC-δ, p-PKC-δ, collagen I, fibronectin and α-SMA western blot images and quantitation in kidneys (n = 5). **G**, **H** Representative images of IHC staining of collagen I, α-SMA, fibronectin, F4/80 and CD45 in kidneys of the three groups (n = 5). Bar = 50 μm. **I** Quantitative data showing IHC staining of F4/80 and CD45 (n = 5). **J** Relative mRNA levels of IL-6, IL-1β, TNF-α, and MCP-1 in kidneys of the three groups (n = 5). **K**, **L** Western blot and quantitative data showing the protein levels of VDAC1, STING, p-TBK1, TBK1, p-p65 and p65 in kidneys of the three groups (n = 5). Data are presented as mean *±* SEM. **P < 0.01, ***P < 0.001.

To investigate the role of PKC-δ in kidney injury, we treated UUO mice with Rottlerin, an inhibitor of PKC-δ, by intraperitoneal injection (Fig. 2A). Hematoxylin and eosin (H&E) and Masson’s trichrome staining indicated that Rottlerin alleviated UUO-induced renal tubule injury and renal interstitial fibrosis (Fig. 2B–D). Rottlerin treatment significantly suppressed PKC-δ phosphorylation in UUO kidneys (Fig. 2E, F). The expression of fibrosis-related proteins such as collagen I, fibronectin and α-SMA in UUO kidneys was attenuated by Rottlerin (Fig. 2E–G). Immunostaining detection revealed macrophage markers F4/80, and leukocyte common antigen CD45 were reduced after Rottlerin treatment in kidneys after UUO (Fig. 2H, I). In addition, the UUO-induced expression of inflammation-related genes such as IL-6, IL-1β, MCP-1 and TNF-α was also decreased in Rottlerin-treated mice (Fig. 2J).

VDAC1 expression was enhanced in the kidney of UUO mice, which was blunted by Rottlerin treatment (Fig. 2K, L). In addition, immunohistochemistry results showed that Rottlerin reduced UUO-induced VDAC1 expression in kidneys (Supplementary Fig. S1A). Next, we evaluated the effect of PKC-δ inhibition on cGAS-STING signaling pathway activation in UUO kidneys. The STING expression, and phosphorylation levels of TBK1 and p65 were increased in UUO kidneys; while these changes were significantly inhibited by Rottlerin (Fig. 2K, L). Meanwhile, immunofluorescence staining indicated that increased coexpression of PKC-δ and VDAC1 in kidneys after UUO, which was abolished by Rottlerin (Supplementary Fig. S1B).

### Inhibition of VDAC1 attenuates UUO-induced renal fibrosis, inflammation and cGAS-STING pathway activation

**Fig. 3:**
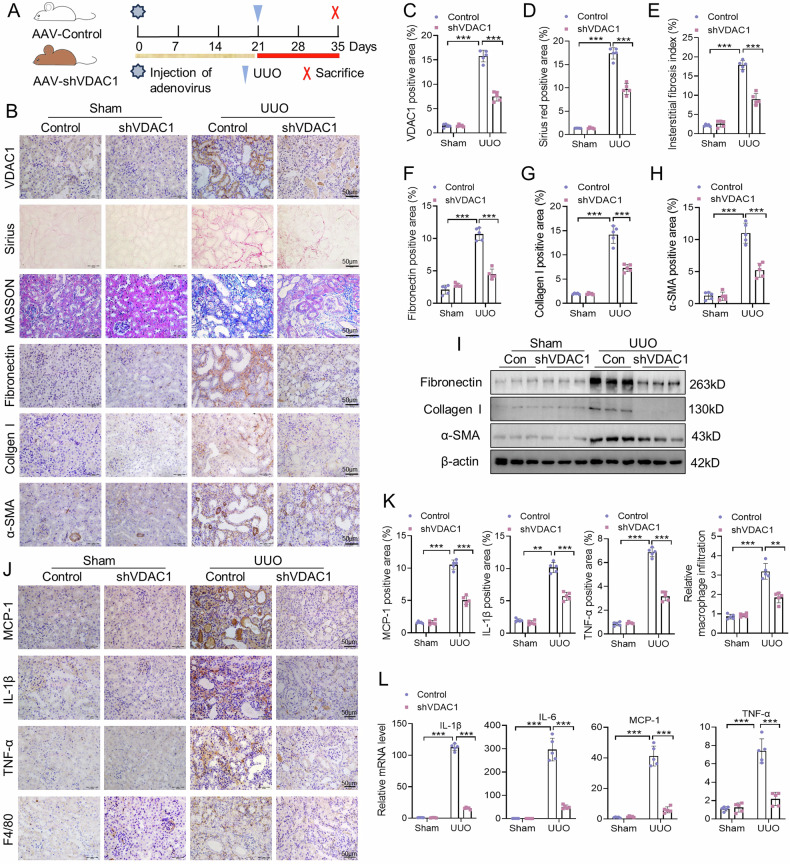
Inhibition of VDAC1 attenuates UUO-induced renal fibrosis, inflammation and cGAS-STING pathway activation. The mice were randomly separated into the Sham+AAV-Control, UUO + AAV-Control, Sham+AAV-shVDAC1, UUO + AAV-shVDAC1 groups. **A** Experimental design: Wild-type C57BL/6 mice were injected with 150 μL of 1.3*10^12^ vg/ml infective units of AAV-control or AAV-shVDAC1 via tail vein before UUO surgery. **B**–**H** Representative images of Sirius red, Masson staining, and IHC staining of VDAC1, fibronectin, collagen I, α-SMA and quantitation in kidneys injected with AAV-Control or AAV-shVDAC1 (n = 5). Bar = 50 μm. **I** Representative western blot images of collagen I, fibronectin and α-SMA in kidneys of the four groups. **J**, **K** Representative images of IHC staining of MCP-1, TNF-α, IL-1β and F4/80 and quantitation in kidneys (n = 5). Bar = 50 μm. **L** Relative mRNA levels of IL-6, IL-1β, TNF-α, and MCP-1 in kidneys (n = 5). Data are presented as mean ± SEM. **P < 0.01, ***P < 0.001.

Next, we evaluated the role of VDAC1 in kidney injury after UUO using AAV-shVDAC1 or AVV-control treatment (Fig. 3A). As shown in Fig. 3B, C, increased VDAC1 expression in kidney after UUO was blocked by AAV-shVDAC1 treatment. Masson’s trichrome staining and Sirius red staining results showed that kidney fibrosis was significantly increased in UUO mice compared with sham controls, while this alteration was attenuated by AAV-shVDAC1 treatment (Fig. 3B, D, E). Meanwhile, absence of VDAC1 suppressed UUO-induced expression of fibronectin, collagen I, and α-SMA in kidneys (Fig. 3B, F–I). In addition, we observed the effect of VDAC1 inhibition on inflammatory responses in kidney after UUO. The expression levels of MCP-1, TNF-α, IL-1β, and F4/80 were significantly higher in AAV-control UUO mice than those in AAV-control sham mice (Fig. 3J, K). However, these alterations were impeded by AAV-shVDAC1 treatment (Fig. 3J, K). Moreover, AAV-shVDAC1 significantly prevented UUO-induced mRNA expression levels of MCP-1, TNF-α, IL-1β, and IL-6 in kidneys of mice (Fig. 3L). Furthermore, we also found that AAV-shVDAC1 significantly suppressed STING expression and phosphorylation levels of TBK1 and p65 in the kidney of UUO mice (Supplementary Fig. S2A–C).

We further explored the effect of VDAC1 pharmacological inhibition on UUO-induced kidney injury in mice. Mice were treated with 20 mg/kg VBIT-12 after UUO surgery (Supplementary Fig. S3A). VBIT-12 significantly depressed VDAC1 expression in kidneys of mice after UUO (Supplementary Fig. S3B, C). Masson’s trichrome staining and Sirius red staining showed that VBIT-12 reduced UUO-induced kidney fibrosis in mice (Supplementary Fig. S3B, C). Similarly, VBIT-12 had the ability to alleviate the expression of collagen I, α-SMA and fibronectin in kidneys after UUO (Supplementary Fig. S3B–D). Consistently, VBIT-12 also effectively attenuated the expression levels of F4/80, MCP-1 and IL-1β in UUO kidneys (Supplementary Fig. S3E, F). RT-qPCR revealed that the mRNA levels of MCP-1, TNF-α, IL-1β, and IL-6 induced by UUO were significantly alleviated by VBIT-12 (Supplementary Fig. S3G). Meanwhile, VBIT-12 inhibited STING expression as well as its downstream signaling molecules, p-TBK1 and p-p65, in UUO kidneys (Supplementary Fig. S4A–C). Thus, these data indicated that VDAC1 is involved in the fibrosis, inflammation, and activation of cGAS-STING pathway in kidneys after UUO.

### STING deficiency alleviates UUO-induced fibrosis and inflammation in mice

**Fig. 4:**
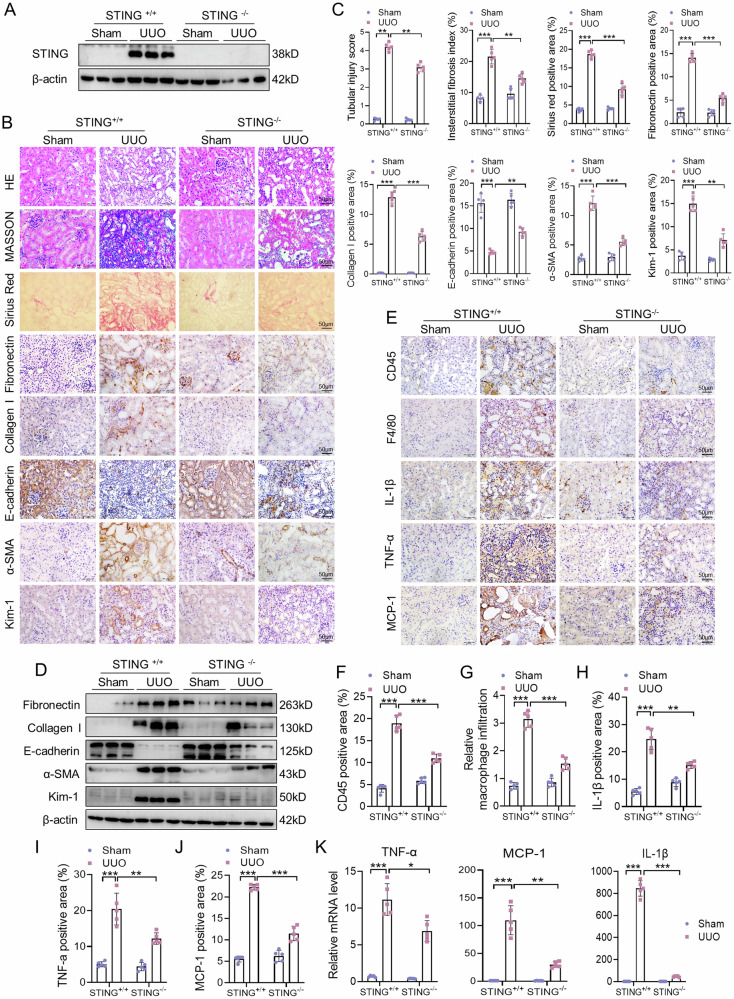
STING deficiency alleviates UUO-induced fibrosis and inflammation in mice. STING^−/−^ mice and their littermates (STING^+/+^) mice were randomly separated into the STING^+/+^+Sham, STING^+/+^ + UUO, STING^−/−^+Sham and STING^−/−^ + UUO groups. **A** Representative western blot images of STING in the kidneys of STING^+/+^ and STING^−/−^ mice after UUO. **B**, **C** Representative images of HE, Masson, Sirius red, and IHC staining of fibronectin, collagen I, E-cadherin, α-SMA and Kim-1 and quantitative analysis in kidneys (n = 5). Bar = 50 μm. **D** Representative western blot images of fibronectin, collagen I, E-cadherin, α-SMA and Kim-1 in kidneys of the four groups. **E**–**J** Representative images of IHC staining of MCP-1, TNF-α, IL-1β, CD45 and F4/80 and quantitative analysis in kidneys (n = 5). Bar = 50 μm. **K** Relative mRNA levels of IL-6, TNF-α, and MCP-1 in kidneys (n = 5). Data are presented as mean ± SEM. *P < 0.05, **P < 0.01, ***P < 0.001.

To investigate the role of cGAS-STING signaling pathway in renal injury, we established a STING knockout (STING^−/−^) mice. The knockout efficiency of STING was confirmed by western blot (Fig. 4A). STING^+/+^ mice showed significant tubular injury after UUO, while this change was ameliorated by STING deletion (Fig. 4B, C). Masson’s trichrome staining and Sirius red staining confirmed that STING^−/−^ mouse kidneys displayed a reduced fibrosis after UUO compared with STING^+/+^ kidneys (Fig. 4B, C). Compared with STING^+/+^ sham mice, the renal expression levels of fibronectin, collagen I, α-SMA, and Kim-1 were significantly increased and expression of E-cadherin was decreased in the STING^+/+^ UUO mice, while these changes were reversed in STING^−/−^ UUO mice (Fig. 4B–D).

We next evaluated the effect of STING deletion on UUO-induced inflammation in kidneys of mice. There was an increase in the numbers of F4/80-positive macrophage and CD45-positive leukocyte infiltration at 14 days after UUO in STING^+/+^ mice (Fig. 4E–G). The numbers of F4/80-positive cells and CD45-positive cells were lower in STING^−/−^ UUO kidneys than in STING^+/+^ UUO kidneys (Fig. 4E–G). Consistent with the reduction in F4/80-positive cells and CD45-positive cells, UUO-induced protein and mRNA expression levels of MCP-1, TNF-α and IL-1β were ameliorated in the kidneys of STING^−/−^ mice (Fig. 4E, H–K).

To further evaluate the effect of cGAS-STING pathway in kidney injury, we treated UUO mice with C-176, a selective STING inhibitor, for 14 days (Supplementary Fig. S5A). We found that C-176 significantly alleviated renal tubular injury and fibrosis, inhibited fibronectin, collagen I and α-SMA expressions, and reversed E-cadherin expression in kidneys of UUO mice (Supplementary Fig. S5B–D). In addition, UUO-induced F4/80-positive macrophage and CD45-positive leukocyte infiltration and expression of proinflammatory factors (MCP-1, TNF-α and IL-1β) were markedly reduced by C-176 treatment (Supplementary Fig. S5E–J). Similarly, C-176 significantly downregulated mRNA levels of MCP-1, TNF-α and IL-1β in kidney after UUO (Supplementary Fig. S5K).

To scrutinize the molecular mechanisms of STING inhibition in kidney injury, we detected STING downstream signaling molecules. Compared with STING^+/+^ sham mice, the phosphorylation levels of TBK1 and p65 were increased in STING^+/+^ UUO mice (Supplementary Fig. S6A–C). The phosphorylation of TBK1 and p65 was lower in STING^−/−^ UUO kidneys than in STING^+/+^ UUO kidneys (Supplementary Fig. S6A–C). In addition, UUO-induced NF-κB p65 translocation from cytoplasm to nuclear was prevented by the ablation of STING (Supplementary Fig. S6D, E). Moreover, UUO-induced phosphorylation of TBK1 and p65 was suppressed by C-176 treatment (Supplementary Fig. S6F, G). These data suggest that STING facilitates the downstream TBK1/NF-κB signaling in kidneys after UUO.

### Inhibition of STING inhibits hypoxia-induced fibrotic and inflammatory responses in HK-2 cells

**Fig. 5:**
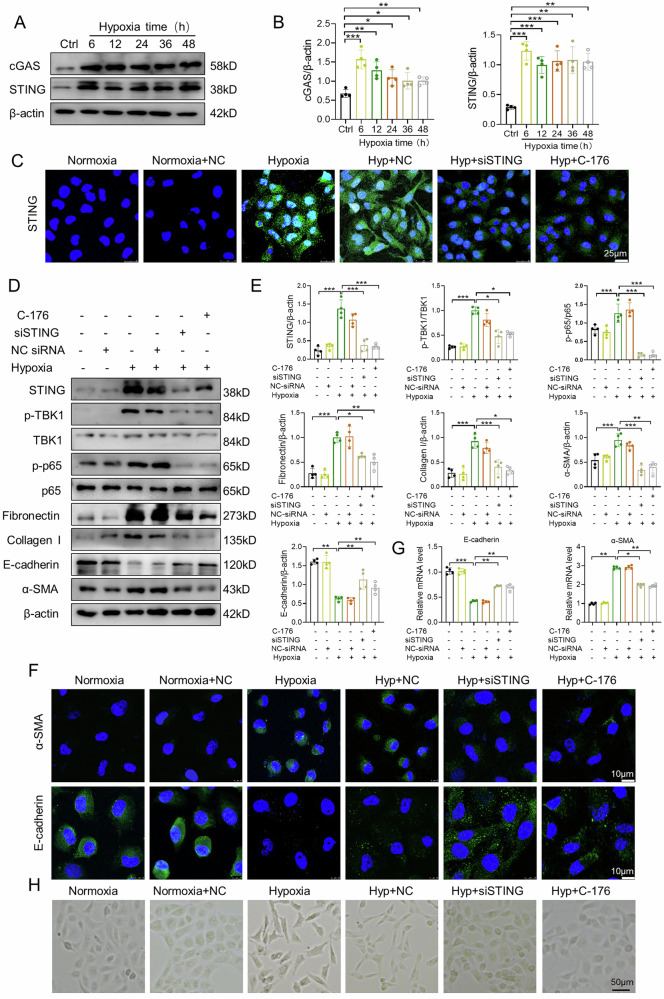
Inhibition of STING inhibits hypoxia-induced fibrotic and inflammatory responses in HK-2 cells. **A**, **B** HK-2 cells were cultured with hypoxia at various time points. Representative western blot images and quantification of cGAS and STING in HK-2 cells (n = 4). **C** HK-2 cells exposed to normoxia or hypoxia were transfected with negative control (NC) siRNA or STING siRNA, or treated with C-176. Representative confocal microscopic images of STING in HK-2 cells. Bar = 25 μm. **D**, **E** Representative western blot images and quantification of STING, p-TBK1, TBK1, p-p65, p65, fibronectin, collagen I, E-cadherin and α-SMA in HK-2 cells (n = 4). **F** Representative confocal microscopic images of E-cadherin and α-SMA in HK-2 cells. Bar = 10 μm. **G** Relative mRNA levels of IL-1β, MCP-1 and TNF-α in HK-2 cells (n = 4). **H** Morphological changes in HK-2 cells were analyzed under an inverted microscope. Bar = 50 μm. NC: negative control, Hyp: hypoxia. Data are presented as mean *±* SEM. *P < 0.05, **P < 0.01, ***P < 0.001.

Hypoxia-induced renal fibrosis is associated with a variety of cellular and molecular mechanisms [34]. We found that the expression of cGAS and STING in HK-2 cells were increased in hypoxia conditions from 6 h to 48 h (Fig. 5A, B). STING siRNA transfection or C-176 treatment significantly inhibited STING expression in HK-2 cells under hypoxia conditions (Fig. 5C–E). Hypoxia-induced phosphorylation of TBK1 and NF-κB p65 was alleviated by transfection of STING siRNA or C-176 treatment (Fig. 5D, E). The expression of fibronectin and collagen I was elevated in HK-2 cells exposed to hypoxia, which was suppressed by STING siRNA or C-176 (Fig. 5D, E). Next, we evaluated the effect of STING on EMT in hypoxia-induced HK-2 cells. Hypoxia resulted in an increase of α-SMA and decrease of E-cadherin, which was restored by STING siRNA or C-176 in HK-2 cells (Fig. 5D–F). In addition, the hypoxia-induced α-SMA mRNA upregulation and E-cadherin mRNA downregulation were reversed by STING siRNA or C-176 (Fig. 5G). The morphological changes induced by hypoxia were also reversed by STING silencing or C-176 (Fig. 5H). Previous studies have demonstrated that inflammation played an important role in renal fibrosis [4, 35]. We found that STING siRNA or C-176 significantly inhibited mRNA expression of proinflammatory factors, including MCP-1, TNF-α, and IL-1β in HK-2 cells exposed to hypoxia (Supplementary Fig. S7).

### VDAC1 mediates hypoxia-induced STING signaling activation, and fibrotic and inflammatory responses in vitro

**Fig. 6:**
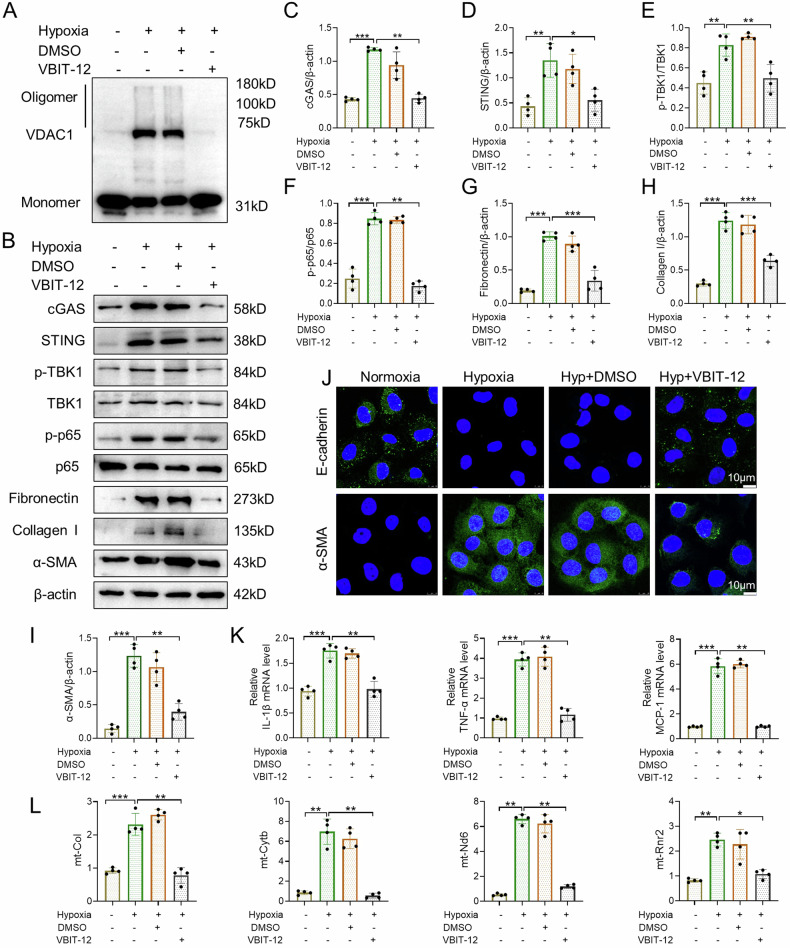
VDAC1 mediates hypoxia-induced STING signaling activation, and fibrotic and inflammatory responses in vitro. **A** Representative western blot image of VDAC1 oligomerization in HK-2 cells. **B**–**I** Representative western blot images and quantification of cGAS, STING, p-TBK1, TBK1, p-p65, p65, fibronectin, collagen I, and α-SMA in HK-2 cells (n = 4). **J** Representative confocal microscopic images of E-cadherin and α-SMA in HK-2 cells. Bar = 10 μm. **K** Relative mRNA levels of IL-1β, TNF-α, and MCP-1 in HK-2 cells (n = 4). **L** qPCR showed the cytosolic translocation of mtDNA (mt-Col, mt-Cytb, mt-Nd6, mt-Rnr2) in HK-2 cells (n = 4). Hyp: hypoxia. Data are presented as mean *±* SEM. *P < 0.05, **P < 0.01, ***P < 0.001.

To further validate the effect of VDAC1 on cGAS-STING pathway activation and cell injury, HK-2 cells were treated with VBIT-12 for 48 h under hypoxia conditions. As shown in Fig. 6A, hypoxia resulted in significant oligomerization of VDAC1 in HK-2 cells, and this alteration was restrained by VBIT-12. VBIT-12 markedly prevented cGAS and STING expressions, and inhibited phosphorylation levels of TBK1 and p65 in HK-2 cells exposed to hypoxia (Fig. 6B–F). In addition, hypoxia-induced expression of fibronectin, collagen I, and α-SMA were alleviated by VBIT-12 (Fig. 6B, G–I). Meanwhile, immunofluorescence showed that increased α-SMA and decreased E-cadherin induced by hypoxia were reversed by VBIT-12 (Fig. 6J). Moreover, hypoxia-induced mRNA expression of MCP-1, TNF-α, and IL-1β in HK-2 cells was remarkably attenuated by VBIT-12 (Fig. 6K). Recent study has shown that voltage-dependent anion channel (VDAC) oligomer pores promote mitochondrial outer membrane permeability and allow mitochondrial DNA (mtDNA) to be released into the cytoplasm, activating the cGAS-STING signaling pathway [36]. Therefore, we examined whether VBIT-12 could restrain the release of mitochondrial DNA into the cytoplasm in HK-2 cells exposed to hypoxia. As shown in Fig. 6L, hypoxia resulted in significant release of mtDNA (mt-Col, mt-Cytb, mt-ND6, and mt-Rnr2), while this change was reduced by VBIT-12 in HK-2 cells. In summary, these data suggest that pharmacological inhibition of VDAC1 eliminate hypoxia-induced fibrotic and inflammatory responses by regulating cGAS-STING signaling activation in HK-2 cells.

### Inhibition of PKC-δ attenuates hypoxia-induced cGAS-STING signaling activation, and fibrotic and inflammatory responses in vitro

**Fig. 7:**
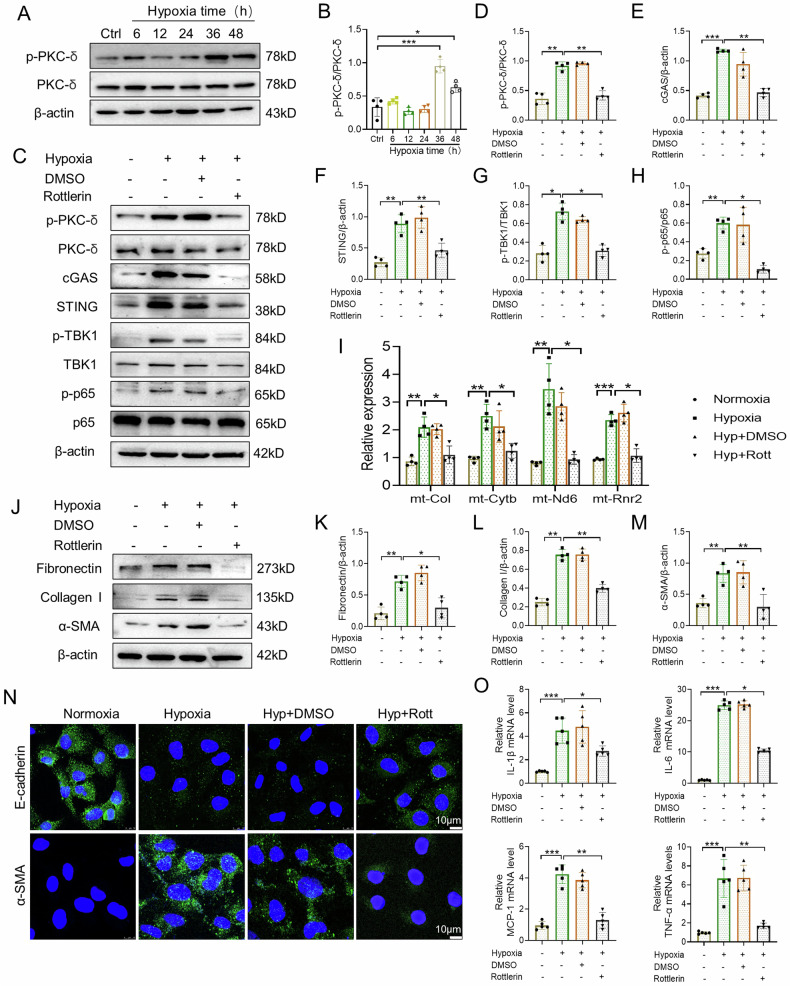
Inhibition of PKC-δ attenuates hypoxia-induced STING signaling activation, and fibrotic and inflammatory responses in vitro. **A**, **B** HK-2 cells were cultured with hypoxia in various time points. Representative western blot images of p-PKC-δ and PKC-δ, and quantification in cell lysate (n = 4). **C**–**H** Representative western blot images and quantification of p-PKC-δ, PKC-δ, cGAS, STING, p-TBK1, TBK1, p-p65, and p65 in HK-2 cells (n = 4). **I** RT-PCR showing the effects of Rottlerin on the cytosolic translocation of mtDNA (mt-Col, mt-Cytb, mt-Nd6, mt-Rnr2) in HK-2 cells (n = 4). **J**–**M** Representative western blot images and quantification of fibronectin, collagen I, and α-SMA in HK-2 cells (n = 4). **N** Representative confocal microscopic images of E-cadherin and α-SMA in HK-2 cells. Bar = 10 μm. **O** Relative mRNA levels of IL-1β, IL-6, TNF-α, and MCP-1 in HK-2 cells (n = 5). Ctrl: control; Hyp: hypoxia; Rott: Rottlerin. Data are presented as mean *±* SEM. *P < 0.05, **P < 0.01, ***P < 0.001.

In order to elucidate the effect of PKC-δ in hypoxia-induced fibrotic and inflammatory responses in vitro, HK-2 cells were treated with Rottlerin. Western blot results indicated that PKC-δ phosphorylation was increased when cells were treated with hypoxia for 36 h and 48 h (Fig. 7A, B). Rottlerin markedly inhibited hypoxia-induced PKC-δ phosphorylation (Fig. 7C, D). Meanwhile, we found that hypoxia-induced expression of cGAS, STING, p-TBK1, and p-p65 was abolished by Rottlerin (Fig. 7C, E–H). In addition, hypoxia-induced release of mtDNA (mt-Col, mt-Cytb, mt-ND6, and mt-Rnr2) was retarded by Rottlerin in HK-2 cells (Fig. 7I). Moreover, Rottlerin significantly inhibited expression levels of collagen I, fibronectin, and α-SMA in HK-2 cells exposed to hypoxia (Fig. 7J–M). Immunofluorescence staining showed that hypoxia-induced increased expression of α-SMA and decreased expression of E-cadherin were restored by Rottlerin (Fig. 7N). Furthermore, Rottlerin significantly reduced mRNA expression of proinflammatory factors, including TNF-α, MCP-1, IL-6, and IL-1β (Fig. 7O). To verify the effect of mtDNA released from mitochondria on activation of cGAS-STING sinaling pathway, dideoxycytidine (ddC), an inhibitor of mitochondrial DNA polymerase γ, was used to treat HK-2 cells. Dideoxycytidine treatment efficiently reduced release of mtDNA and suppressed expression of cGAS, STING, p-TBK1, and p-p65 in HK-2 cells exposed to hypoxia (Supplementary Fig. S8A–C). Taken together, these results suggest that inhibition of PKC-δ prevents activation of cGAS-STING signaling pathway via preventing mtDNA release in hypoxia-treated HK-2 cells.

### PKC-δ binds to VDAC1 and promotes its oligomerization to increase mitochondrial impairment in HK-2 cells exposed to hypoxia

**Fig. 8:**
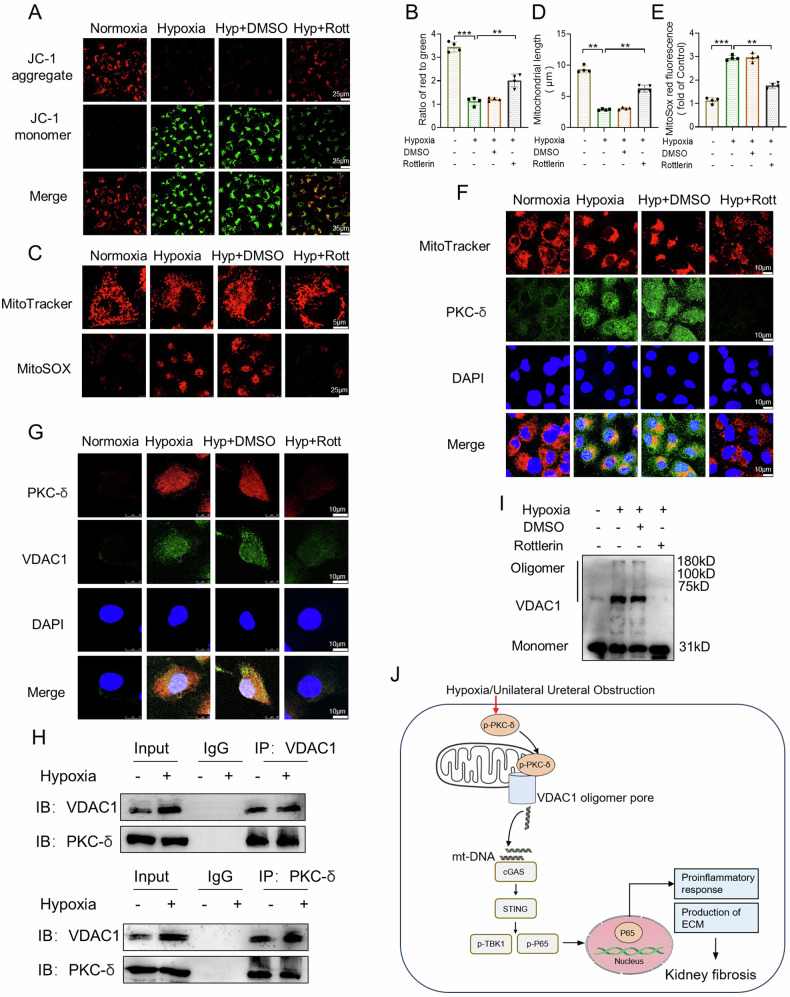
PKC-δ binds to VDAC1 and promotes its oligomerization to increase mitochondrial impairment in HK-2 cells exposed to hypoxia. **A**, **B** Representative images and quantification of JC-1 staining showing the effect of Rottlerin on mitochondrial membrane potential (MMP) in HK-2 cells exposed to hypoxia (n = 4). Bar = 25 μm. **C**–**E** Representative images and quantification of MitoTracker and MitoSOX showing the effects of Rottlerin on the mitochondria fragmentation and mtROS generation in HK-2 cells exposed to hypoxia (n = 4). Bar = 5 μm (MitoTracker) and Bar = 25 μm (MitoSOX). **F** Representative images of MitoTracker and PKC-δ costaining in HK-2 cells. Bar = 10 μm. **G** Representative images showing the effect of Rottlerin on colocalization of PKC-δ and VDAC1 in HK-2 cells exposed to hypoxia. Bar = 10 μm. **H** Co-immunoprecipitation was used to detect the interaction of PKC-δ and VDAC1. **I** Representative western blot image of VDAC1 oligomerization in HK-2 cells. **J** Schematic model: PKC-δ/VDAC1/cGAS-STING-mediated inflammation and fibrosis in the development of obstructive nephropathy. Hyp: hypoxia; Rott: Rottlerin. Data are presented as mean *±* SEM. **P < 0.01, ***P < 0.001.

To elucidate the mechanism by which PKC-δ regulates VDAC1 oligomerization in HK-2 cells, we evaluated its effect on the mitochondrial changes and the relationship between PKC-δ and VDAC1. A recent study has shown that VDAC oligomer pores promote mitochondrial outer membrane penetration [37]. Firstly, we measured mitochondrial membrane potential (MMP) using the JC-1 fluorescent probe. There was a significant decrease of MMP in HK-2 cells exposed to hypoxia, while Rottlerin could restore this change (Fig. 8A, B). Next, we evaluated mitochondrial morphology using the MitoTracker Red fluorescent probe. As shown that in Fig. 8C, D, hypoxia-induced decrease in the average length of mitochondria was reversed by Rottlerin treatment. In addition, MitoSOX Red staining revealed hypoxia-induced mitochondrial ROS production was significantly inhibited by Rottlerin (Fig. 8C, E). The results of MitoTracker Red and PKC-δ co-staining showed that PKC-δ expression in mitochondria was elevated in HK-2 cells under hypoxia conditions, while this change was markedly blunted by Rottlerin (Fig. 8F). Moreover, immunofluorescence double staining showed that there was an increased colocalization of PKC-δ and VDAC1 in HK-2 cells exposed to hypoxia (Fig. 8G). As expected, Rottlerin could reduce PKC-δ and VDAC1 colocalization induced by hypoxia (Fig. 8G). Furthermore, PKC-δ and VDAC1 were separately immunoprecipitated in HK-2 cells exposed to hypoxia. Both PKC-δ and VDAC1 were detected in their individual immunoprecipitated complexes (Fig. 8H). Interestingly, we found that hypoxia-induced oligomerization of VDAC1 was alleviated by Rottlerin (Fig. 8I). Collectively, these findings suggest that PKC-δ is involved in mitochondrial impairment by direct binding with VDAC1, and promoting its oligomerization in hypxia-induced HK-2 cells.

## Discussion

It has been documented that PKC-δ, one of the key subtypes of PKC family, plays an important regulatory role in cell proliferation and differentiation, survival and death, movement and migration [38]. Current understanding of pathological function of PKC-δ in the fibrotic diseases was very limited. This study discovered that PKC-δ expression was upregulated in kidneys, especially in tubules, in both human patients with CKD and mouse models of kidney fibrosis. Rottlerin, a PKC-δ inhibitor, significantly attenuated renal inflammation and fibrosis in UUO mice. Mechanistically, PKC-δ activation resulted in mitochondrial membrane VDAC1 oligomerization, followed by the release of mtDNA, and subsequently activated cGAS-STING signaling pathway, and promoted inflammatory responses which contributed to the renal fibrosis. These data unveil that inhibition of PKC-δ may have a beneficial effect on renal interstitial fibrosis.

Previous studies have shown that PKC-δ contributed to tubular cell injury associated with proteinuria [13], and acute kidney injury induced by myoglobin [12], vancomycin [39], and cisplatin [11]. In addition, PKC-δ knockout alleviated albuminuria, podocyte apoptosis, and renal mRNA expression of TGFβ, VEGF, and collagen IV in diabetic mice [40]. Therefore, these studies suggest that PKC-δ may play an important role in acute tubular injury and diabetic kidney disease. However, the contribution of PKC-δ to kidney fibrosis is not well known. Epithelial to mesenchymal transition (EMT) is thought to be an important mechanism in the development of organ fibrosis, including renal fibrosis [41–43]. Previous study demonstrated that inhibition of PKC-δ prevented angiotensin II-induced proliferation and collagen accumulation in cardiac fibroblasts [44]. PKC-δ mediated advanced oxidation protein products-induced EMT in intestinal epithelial cells [45]. Meanwhile, PKC-δ inhibition suppressed collagen I biosynthesis and mRNA expression of collagen I and collagen III in normal and systemic sclerosis dermal fibroblasts [46]. In present study, we provide evidence that PKC-δ plays a role in UUO-induced kidney fibrosis. PKC-δ expression was upregulated in kidney after UUO 7 and 14 days in mice accompanied by the exacerbation of renal fibrosis, and Rottlerin, a pharmacologic inhibitor of PKC-δ, suppressed UUO-induced fibrosis in mice. In addition, Rottlerin also ameliorated hypoxia-induced EMT in HK-2 cells. These data suggest that PKC-δ activation is involved in renal fibrosis.

Accumulating evidence has shown that inflammation plays a crucial role in the kidney fibrosis [35]. Infiltration of macrophage and induction of various proinflammatory cytokines such as IL-6, IL-1β, IL-18, chemokine (C-C motif) ligand 2, and TNF-α were associated with kidney fibrosis [47–49]. The contribution of macrophages to kidney fibrosis is well documented [4]. Neutralization of IL-6 using IL-6 neutralizing antibody suppressed kidney fibrosis and collagen deposition in diabetic mice [50]. Etanercept, an inhibitor of TNF-α, significantly attenuated kidney fibrosis and inflammation in a mouse model of aristolochic acid nephropathy [51]. Activation of NF-κB contributed to fibroblast activation and renal fibrosis, as inhibition of NF-κB activation in interstitial fibroblasts attenuates renal fibrogenesis [52]. Our previous study has demonstrated that NLRP3 deletion alleviated kidney fibrosis via reducing proinflammatory cytokines IL-1β and IL-18 release in diabetic mice [53]. Although the profibrotic action of PKC-δ has not been investigated, PKC-δ promotes inflammatory cell infiltration and/or exerts a proinflammatory effect has been reported. PKC-δ mediated lipopolysaccharide (LPS)-induced microglia activation [54]. Rottlerin treatment reduced cytokines expression in microglia exposed to LPS [54]. Blockade of PKC-δ inhibited activation of transcription factors NF-κB p65 and AP-1 c-jun, and reduced MCP-1 levels in both pancreas and plasma, as well as infiltration of neutrophils in lung in caerulein-induced acute pancreatitis mice [55]. In this study, we found that Rottlerin treatment significantly inhibited infiltration of macrophage and leukocyte, and expression of inflammation-related genes such as IL-6, IL-1β, MCP-1 and TNF-a in kidneys after UUO. Moreover, hypoxia-induced NF-κB activation and mRNA expression of TNF-α, MCP-1, IL-6, and IL-1β were suppressed by Rottlerin. These results suggest that blockage of PKC-δ prevents renal fibrosis by reducing inflammation.

Recent studies demonstrated that activation of cGAS-STING signaling pathway contributed to folic acid or hypoxia-induced renal fibrosis [27, 34]. In present study, we showed that cGAS-STING signaling pathway was activated in kidneys after UUO, and STING deletion or C-176 alleviated UUO-induced inflammation and fibrosis. In addition, hypoxia resulted in activation of cGAS-STING signaling pathway in HK-2 cells. Blockage of STING by C-176 or STING siRNA relieved hypoxia-induced fibrotic responses and mRNA expression of proinflammatory cytokines. Interestingly, activation of cGAS-STING signaling pathway in UUO kidney and HK-2 cells exposed to hypoxia was suppressed by Rottlerin treatment. These data suggest that inhibition of PKC-δ prevents renal fibrosis via suppressing cGAS-STING signaling pathway activation.

The cGAS-STING pathway is a key component of the human innate immune system in sensing abnormal presence of DNA in the cytoplasm [56]. Recent studies documented that mitochondrial damage and mtDNA released into cytoplasm, further activated cGAS-STING signaling pathway [27, 57–59]. Cytoplasmic mtDNA released from mitochondria in damaged tubule cells caused inflammation by triggering cGAS-STING signaling pathway in acute kidney injury induced by cisplatin or hypoxia/reoxygenation [23]. In our experiment, we found that hypoxia resulted in activation of cGAS-STING signaling, accompanied by mtDNA release from mitochondria into cytoplasm in HK-2 cells. In addition, pretreatment with ddC, an inhibitor of mitochondrial DNA polymerase γ, reduced cytoplasmic mtDNA level and activation of cGAS-STING signaling pathway in HK-2 cells under hypoxia conditions. Previous study has shown that renal tubular cells were hypoxic in UUO kidneys [60]. Taken together, these data suggest that hypoxia mediates UUO-induced activation of cGAS-STING signaling pathway via triggering mtDNA release in injury kidney.

A recent study demonstrated that mitochondrial pores formed by VDAC oligomers trigger mtDNA release into the cytosol in live cells [30]. MtDNA could interact with three positively charged residues at the N-terminus of VDAC1, which enhanced VDAC1 oligomerization and facilitated mtDNA release [30]. VBIT-12 inhibited VDAC1 overexpression and oligomerization, and mtDNA release in CT-26 cells treated with either dextran sulfate sodium (DSS) or H_2_O_2_ [61]. Meanwhile, VDAC1siRNA significantly decreased H_2_O_2_-induced release of mtDNA in CT-26 cells [61]. In this study, the expression of VDAC1 was elevated in kidney after UUO, and blockage of VDAC1 attenuated UUO-induced renal fibrosis, inflammation and cGAS-STING pathway activation. In vitro, hypoxia resulted in VDAC1 oligomerization, mtDNA release and activation of cGAS-STING signaling pathway, which were retarded by VBIT-12 treatment. Collectively, these data indicate that VDAC1 oligomerization is involved in mtDNA release and activation of cGAS-STING signaling pathway in the progression of renal fibrosis.

Collecting evidence has shown that PKC-δ is localized in various subcellular fractions, including mitochondria, nucleus, and endoplasmic reticulum [62]. Previous studies have demonstrated that PKC-δ and its mitochondrial translocation contributed to mitochondrial dysfunction [63, 64]. The mitochondrial localization of PKC-δ was involved in the regulation of mitophagy [9, 10]. In our study, PKC-δ was found colocalization with mitochondria in HK-2 cells following hypoxia. In addition, our immunofluorescence double staining and co-IP results demonstrated that PKC-δ interacted with VDAC1 in the mitochondria of HK-2 cells exposed to hypoxia. Interestingly, hypoxia-induced mitochondrial translocation of PKC-δ, mitochondrial dysfunction, mtROS production, and VDAC1 oligomerization were alleviated by Rottlertin treatment in HK-2 cells. Therefore, these data suggest that the interaction between PKC-δ and VDAC1 triggers VDAC1 oligomerization, and mtDNA release.

In summary, our study delineated the role and the underlying mechanism of PKC-δ in UUO-induced nephropathy. After UUO, PKC-δ activation to promote VDAC1 oligomerization, mtDNA release, and activation of cGAS-STING signaling pathway, which contribute to inflammation and fibrosis in kidneys (Fig. 8J). Therapeutic strategies targeting PKC-δ/VDAC1/cGAS-STING-mediated inflammation and fibrosis may prove useful for progression of CKD.

## Materials and methods

### Animal protocols

Heterozygous STING knockout (STING^+/−^) mice aged 8-10w and weighing approximately 20-24 g were purchased from the Cyagen Biosciences Inc (Suzhou, China). Homozygotes STING knockout (STING^−/−^) male mice and their littermates (STING^+/+^) mice after breeding homozygotes pairs were used for experiments. The genotyping of the STING^−/−^ mice at the age of 2-3 weeks was identified by PCR amplication of DNA isolated from tail tissue with the following primers:

Forward: 5’-ACCTGGTTTATGGAGGCTGGTAG-3’;

Reverse: 5’-AATGTTCCTTCTCCCTCTCGCC-3’.

The PCR product for mutant bind (STING^−/−^) is 6097 bp, and that of WT mice is 495 bp.

### Mice model

Mouse unilateral ureteral obstruction (UUO) model was established as described following. The hair was removed from the back of the mice after anesthetized by inhalation of 4% isoflurane. The mice were placed in the prone position on the operating table and the surgical area was sterilized. An oblique incision was made in the back of the mouse, parallel to the costal arch, exposed the inferior renal pole and the upper ureter, and the left ureter was ligated with No. 6 silk thread. Wild-type (WT) littermates which underwent a sham operation were used as control subjects. Sham mice and/or UUO mice were treated with Rottlerin (10 mg/kg), C-176 (750 nmol per mouse), or VBIT-12 (20 mg/kg) (Supplementary Table S2) daily by intraperitoneal injection.

Ischemia/reperfusion (I/R)-induced AKI mouse model was performed by exposing bilateral kidneys. Kidney ischemia was performed for 30 min by clamping the renal pedicle through a lateral ventral incision, the arterial clips were released for reperfusion. The sham mice underwent the same procedure except that the kidney pedicle was not clamped.

The mice were housed in a specific pathogen free facility. For all studies, mice were housed under controlled environmental conditions under a 12-h light/dark cycle and allowed ad libitum access to rodent chow and water.

### Adeno-associated viruses delivery

The 8-week-old age C57BL/6J wild type (WT) male mice were treated with adeno-associated virus 9 carrying the shVDAC1 for interference of VDAC1 gene. Adeno-associated virus 9 vectors (AAV9) carrying VDAC1 interference sequences (AAV-shVDAC1-EGFP, 1.3*10^12^ vg/ml) and its negative control (AAV-Control-EGFP) were designed by Hanheng Biotechnology Company (Shanghai, China). Each mouse was injected with 100 ul 1.3*10^12^ vg/ml AAV9 carrying the shVDAC1 (AAV-shVDAC1-EGFP) or negative control (AAV-EGFP) through tail. UUO or Sham operation was performed at 3 weeks after adeno-associated virus injection.

### Clinical specimens

Renal biopsy residual samples were recruited in the Second Hospital of Hebei Medical University. According to the principles of the Declaration of Helsinki, all human studies were conducted with informed consent of patients and approved by the Medical Ethics Committee of Hebei Medical University (No. 2021033). In this study, twenty five renal biopsy samples from adults were obtained. Including 5 normal renal tissue samples from the distal kidney after local tumor resection, 5 patients with type 2 diabetes mellitus with nephropathy, 5 patients with IgA nephropathy, 5 patients with focal segmental glomerular sclerosis and 5 patients with Lupus nephritis V.

### Histology and immunohistochemical (IHC) staining

Mouse kidney tissues were fixed in 4% paraformaldehyde for 48 h, dehydrated with gradient alcohol, embedded in paraffin, and then sectioned into slices about 3 μm, baked and removed wax. Haematoxylin Eosin (H&E), Sirius Red (Supplementary Table S2), and Masson’s trichrome staining were performed according to a standard protocol. Damaged tubular cells were evaluated as described previously [65]. Lesions were graded on a scale from 0 to 4: 0, normal; 1, <25% damage; 2, 25–50% damage; 3, 50–75% damage; 4, >75% damage. Immunohistochemical staining was performed with SP kit according to the instruction. The paraffin section was repaired with Tris-EDTA (PH = 9.0) repair solution for 8 min, then treated with endogenous peroxidase blockers, and incubated goat serum about 30 min at 37 °C. The kidney sections were incubated with primary antibodies overnight at 4 °C, followed by incubation with appropriate amount of enzyme-labeled sheep anti-mouse/Rabbit IgG polymer (Zhongshan Jinqiao Biotechnology, Beijing, China) for 40 min at 37 °C, and detected with DAB kit (ZhongshanJinqiao Biotechnology, Beijing, China) for 1-2 min. Olympus microscope (Olympus, BX71, Tokyo, Japan) was used to capture the images. Quantitative analysis of positive staining was used by ImageJ software (NIH).

### Cell culture and treatment

The human tubular cell line HK-2 (purchased from China Centre for Type Culture Collection) was cultured in DMEM-F12 (1:1) medium (Gibco, USA) supplemented with 100 U/mL penicillin (Gibco, USA) and 10% fetal bovine serum (CellMax, China) in 5% CO_2_ and 20.9% O_2_ air at 37 °C. Cells attained 70–80% convergence in normal conditions (5% CO_2_ and 20.9% O_2_) and then grew in a medium without serum under hypoxic conditions (5% CO_2_, and 1% O_2_). HK-2 cells were transfected with siSTING and disrupted RNA (Negative control, NC) using transfection reagent (GenePharma, Shanghai, China) according to the manufacture’s protocols. And HK-2 cells were treated with C-176 (3 μM), Rottlerin (1 μM), VBIT-12 (20 μM) or dideoxycytidine (ddC, 40 μg/ml) (Supplementary Table S2)respectively under normoxia or hypoxia conditions.

### Western blot

Total proteins from HK-2 cells and renal tissues were extracted using the radio immunoprecipitation assay (RIPA) lysis buffer (BestBio, Shanghai, China). Nuclear protein and cytoplasma protein were extracted using Nuc-Cyto-Mem Preparation Kit (Applygen Biotechnology, Beijing, China). The protein concentration was measured using the BCA protein kit (Solarbio, Beijing, China). Equal amounts of protein were separated by SDS-PAGE and transferred onto polyvinylidene difluoride (PVDF) membranes (Millipore, MA, USA). And then, membranes were incubated with primary antibodies overnight at 4 °C (Supplementary Table S1). Image acquisition was performed using Amersham Imager 600 (General Electric Company, USA) or Tanon 4800 (Beijing, China). Band densitometry was analyzed using ImageJ software (NIH).

### Quantitative real-time PCR (qPCR)

Total RNA was obtained by TRIzol reagent (TIANGEN, Beijing, China) from HK-2 cells and kidney tissues, and cDNA was prepared using MonScript™ RTIII (Monad, Guangdong, China) according to the instructions. The primers used were listed in Supplementary Table 3. Real-time PCR was performed in a 96-well optical reaction plate using MonAmp^TM^ ChemoHS qPCR Mix (Monad, China). Quantitative PCR reactions were performed on Agilent Mx3000P QPCR Systems (Agilent, CA, USA). The experiment was repeated three times, and the relative gene expression levels were calculated using the 2^−ΔΔCt^ method.

### Mitochondrial DNA release assay

Mitochondrial DNA (mtDNA) in the cytosolic fraction was isolated from HK-2 cells as described previously [27, 66]. HK-2 cells were lysed by mild detergent (0.1% NP-40) and incubated on ice for 15 min. Lysates were centrifuged at 13,000 rpm for 15 min at 4 °C. Cytosolic mtDNA from supernatant cytosolic fraction was purified using a DNeasy Blood & Tissue Kit (QIAGEN) according to manufacturer’s instructions. Quantitative PCR was employed to measure mtDNA using SYBR Premix Ex TaqTM II (Takara, Japan) on Agilent Mx3000P QPCR Systems (Agilent, CA, USA). The data were normalized and analyzed using the 2^−ΔΔCt^ method. Primer sequences for mtDNA determination were shown in Supplementary Table 3.

### Immunofluorescence staining

HK-2 cells were grown and stimulated on a slide in a six-well plate, washed with PBS and fixed with 4% paraformaldehyde for 30 min at room temperature. Cells were treated with 0.3% Triton X-100/PBS about 8–10 min at 37 °C, then incubated goat serum about 30 min at 37 °C. Paraffin-embedded sections were dewaxed, hydrated and repaired with Tris-EDTA (PH = 9.0). HK-2 cells and sections were incubated with primary antibodies overnight at 4 °C. Then the FITC conjugated goat anti-rabbit IgG (Santa Cruz Biotechnology, Santa Crux, CA) was incubated for 2 h at 37 °C. The nucleus was stained with DAPI (SouthernBiotech, America) and the stained slides were imaged using a laser confocal microscope (SP8, Leica, Germany).

### Immunofluorescence double staining

Frozen sections of kidney tissue (6 um) were fixed with cold acetone for 15 min and washed with PBS, incubated goat serum about 30 min at 37 °C. The sections were incubated with primary antibodies for PKC-δ, AQP1, calbindin-D28k, and VDAC1 in PBS overnight at 4 °C. HK-2 cells were grown and stimulated on a slide in a six-well plate, washed with PBS and fixed with 4% paraformaldehyde for 30 min at room temperature. Treatment with 0.3% Triton X-100/PBS about 8–10 min at 37 °C, then incubated with goat serum about 30 min at 37 °C. The slides were incubated with primary antibodies for PKC-δ, and VDAC1 in PBS at 4 °C overnight. Then the cells or sections were incubated with goat anti-rabbit/mouse IgG conjured to Alexa Fluor 488/594 (Abcam, Cambridge, MA, USA) for 2 h at 37 °C and the nucleus was stained using DAPI. The stained samples were viewed using a confocal microscope (SP8, Leica, Germany).

### MitoTracker Red and immunofluorescence staining

HK-2 cells were stained with MitoTracker™ Red CMXRos (200 nM, Thermo Fisher Scientific, US) at 37 °C for 30 min, washed with PBS and fixed with cold 4% paraformaldehyde for 30 min, permeated with 0.3% Triton X-100 for 8 min at 37 °C. Goat serum was incubated at 37 °C for 30 min, and then incubated with anti-PKC-δ primary antibody in a wet chamber at 4 °C overnight. Then the FITC conjugated goat anti-rabbit IgG (1:100; Santa Cruz Biotechnology, Santa Crux, CA) was incubated for 2 h at 37 °C before DAPI staining. The stained samples were viewed using a confocal microscope (SP8, Leica, Germany). To evaluated the mitochondrial morphology, the average length of mitochondria was measured after MitoTracker Red staining [67].

### Measurement of mtROS

Mitochondrial reactive oxygen species (mtROS) was detected by MitoSox Red (Thermo Fisher Scientific, US) according to the manufacturer’s instructions. HK-2 cells were incubated with MitoSox Red at a final concentration of 5 μM at 37 °C for 30 min. The stained cells washed three times with Hank’s balanced salt solution (HBSS) and observed using a confocal microscope (Leica, Germany). The mitochondrial ROS intensity was quantified using the software Image-Pro Plus 6.0.

### Mitochondrial transmembrane potential (MMP)

Mitochondrial membrane potential was detected with the JC-1 kit (C2006, Beyotime, China) according to the manufacturer’s instructions. In brief, added 1 ml JC-1 working fluid to the holes of each six-well plate, incubated at 37 °C for 30 min, and washed with JC-1 liquid buffer in a dark environment for 3 times. 2 ml cell culture medium was added. The stained samples were viewed using a confocal microscope (Leica, Germany). The MMP values were expressed as the ratio of red to green fluorescence levels.

### Coimmunoprecipitation assay

HK-2 cells were washed with ice-cold PBS and lysed with RIPA buffer. After centrifugation at 12000 rpm for 30 minutes at 4 °C, the protein A-Agarose (Santa Cruz, Dallas, TX) were incubated with anti-PKC-δ (1909 mg/mL) or anti-VDAC1 (1000 μg/ml) with continuous shaking for 8 h at 4 °C. After washing the IP complexes, the beads were boiled with 1× buffer for 7 min, and then using immunoblot analysis.

### VDAC cross-linking assay

Cells were treated with the hypoxia as above, collected, washed with PBS, pH 8.3, and then were incubated with EGS (100 mM, 40 min, 30 °C). To remove excess crosslinker, 1.5 M Tris HCl (pH 7.8) was added to a final concentration of 20 mM, incubated at room temperature for 5 min, and then centrifuged at 10000 × *g* for 5 min. The samples were analyzed by SDS-PAGE with anti-VDAC1.

### Statistics

Data are expressed as Mean ± standard error (SEM). The results from at least three independent experiments. Statistical analysis was performed using one-way (ANOVA) analysis of variance. *P* < 0.05 was defined as statistically significant.

### Supplementary information

Supplementary information

Original western blots

## Data Availability

The original data for Western Blot, as well as the prime sequence of genes are available in the supplementary materials.
