# Supplementary material for: Inhibition of PKC-δ retards kidney fibrosis via inhibiting cGAS-STING signaling pathway in mice
Source: Cell Death Discov. 2024 Jul 7;10:314. doi: 10.1038/s41420-024-02087-z (PMC11228024; doi:10.1038/s41420-024-02087-z)
Supplement: Supplementary file 1 — Supplementary information [file 41420_2024_2087_MOESM1_ESM.pdf]

SUPPLEMENTAL MATERIALS

Inhibition of PKC-δ retards kidney fibrosis via inhibiting cGAS-STING pathway in mice

Dongyun Wang[1,2], Yue Li[1], Guiying Li[3], Mengyu Liu[1], Zihui Zhou[1,2], Ming Wu[1,2], Shan Song[1,2], Yawei Bian[1,2], Jiajia Dong[1,2], Xinran Li[1,2], Yunxia Du[1,2], Tao Zhang[2,4], Yonghong Shi[1,2]*

[1] Department of Pathology, Hebei Medical University, Shijiazhuang, 050017, China

[2] Hebei Key Laboratory of Kidney Disease, Shijiazhuang, 050017, China

[3] Department of Nephrology, Affiliated Hospital of Hebei University of Engineering, Handan, 056000, China

[4] Department of Nephrology, Third Hospital of Hebei Medical University, Shijiazhuang, 050051, China.

* Corresponding authors at: Department of Pathology, Hebei Medical University, No. 361 East Zhongshan Road, Shijiazhuang, 050017, PR China.

Yonghong Shi

Tel: +86 311 86266647

E-mail address: yonghongshi@163.com (Y. Shi).

Supplementary Figures

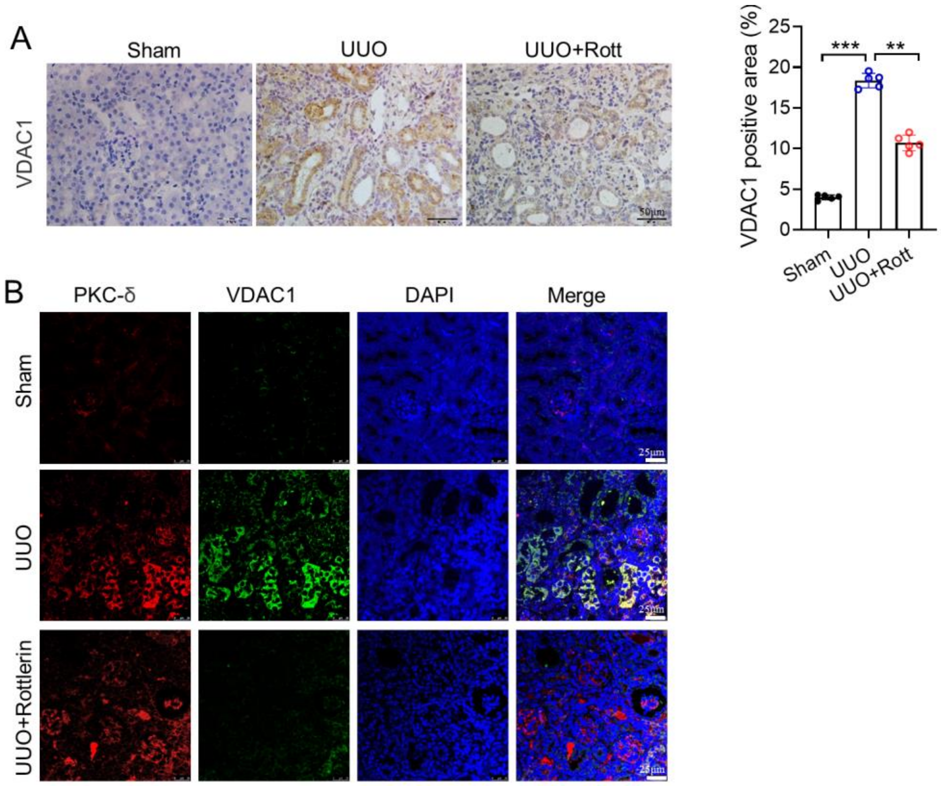

Fig. S1 Rottlerin inhibited UUO-induced VDAC1 expression. A Representative images of VDAC1 IHC staining and quantitation in kidneys of the three groups (n = *5*). Bar = 50 μm. **B** Costaining of PKC-δ and VDAC1 in the kidneys. Bar = 25 μm. Data are presented as mean ± SEM. **P < 0.01, ***P < 0.001.

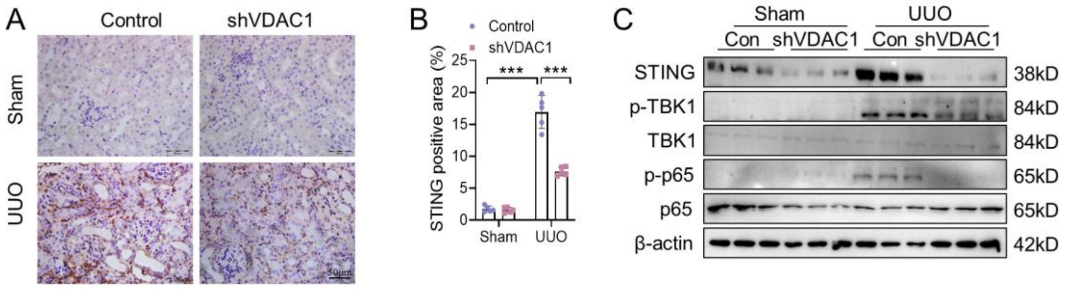

Fig. S2 Inhibition of VDAC1 attenuates UUO-induced cGAS-STING pathway activation. A, B Representative images of IHC staining of STING and quantitation in kidneys of the four groups (n=5). Bar = 50 μm. **C** Representative Western blot images of STING, p-TBK1, TBK1, p-p65, and p65 in kidneys of the four groups. Data are presented as mean ± SEM. ***$P < 0.001$.

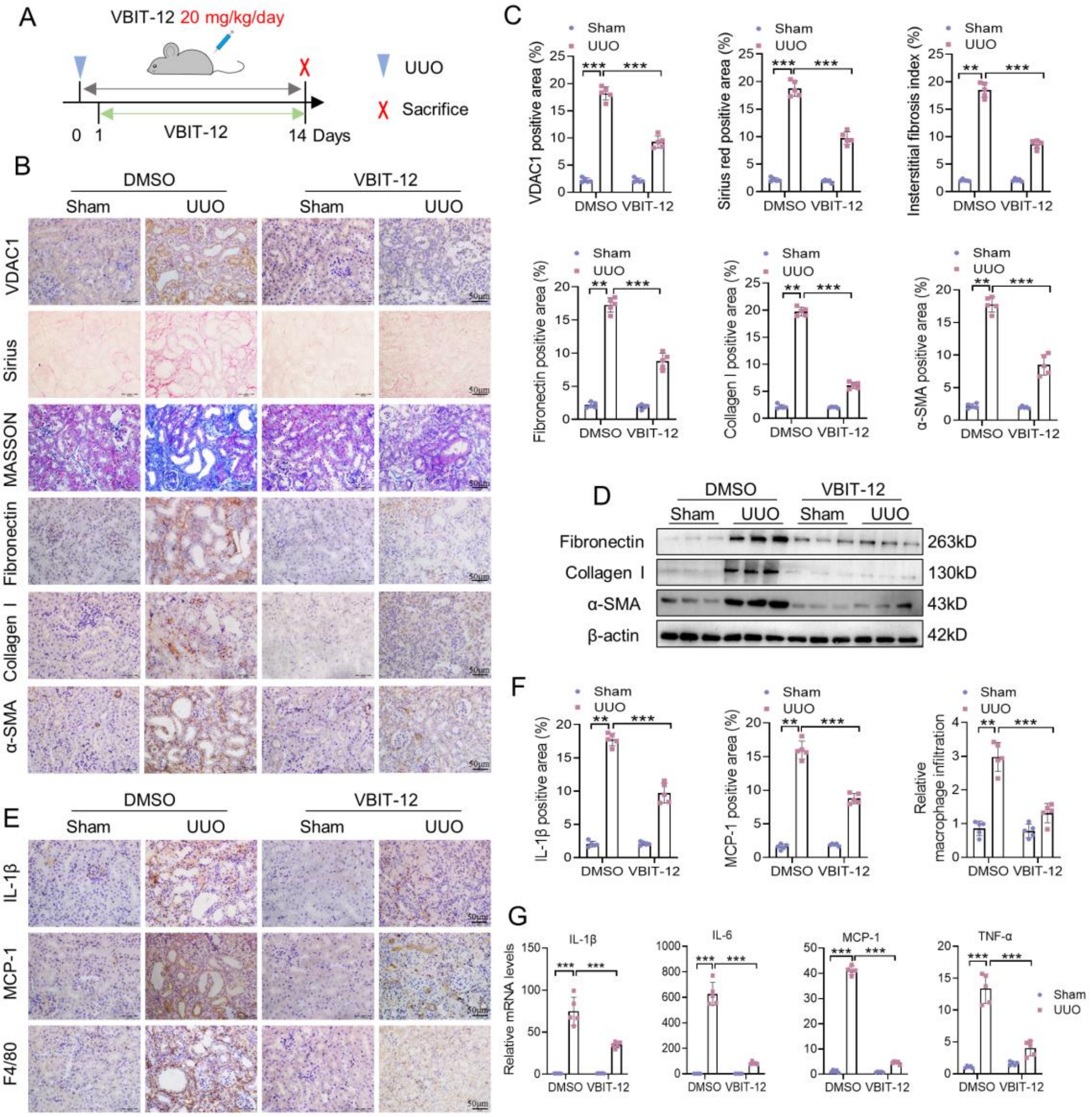

Fig. S3 VBIT-12 alleviates the inflammation and fibrosis in UUO kidneys. The mice were randomly separated into the Sham+DMSO, UUO+DMSO, Sham+VBIT-12 and UUO+VBIT-12 groups. **A** Schematic diagram of the experimental design: wild-type C57BL/6 mice were performed UUO surgery and treated with VDAC1 inhibitor (VBIT-12) for 13 days. The gray left and right double arrows indicates 14 days after UUO surgery, the green arrowheads indicate the injections of VBIT-12 (20 mg/kg/Day). **B-C** Representative images of Sirius red, Masson and IHC staining of VDAC1, fibronectin, collagen I, and α-SMA and quantitation in kidneys of the four groups (n=5). Bar = 50 μm. **D** Representative western blot images of collagen I, fibronectin, and α-SMA in kidneys. **E-F** Representative images of IL-1β, MCP-1, and F4/80 IHC staining and quantitation in kidneys of the four groups (n=5). Bar = 50 μm. **G** Relative mRNA levels of IL-1β, IL-6, TNF-α, and MCP-1 in kidneys of the four groups (n=5). Data are presented as mean ± SEM. ***P* < 0.01, ****P* < 0.001.

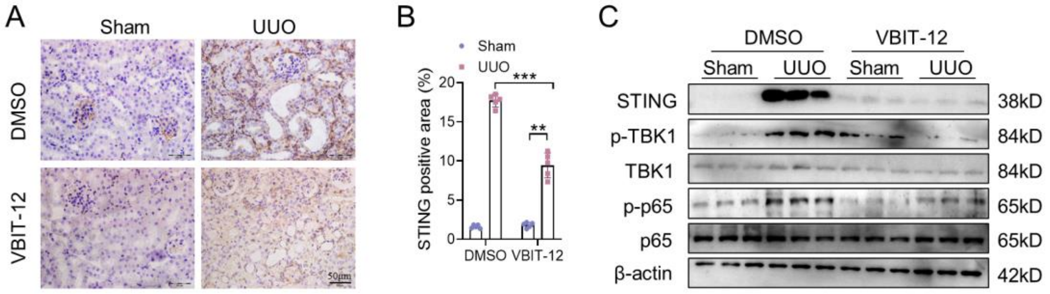

Fig. S4 VBIT-12 attenuates UUO-induced activation of cGAS-STING signaling pathway. A, B Representative images of STING IHC staining and quantitation in kidneys of the four groups (n = 5). Bar = 50 μm. **C** Representative western blot images of STING, p-TBK1, TBK1, p-p65, and p65 in kidneys of the four groups. Data are presented as mean ± SEM. $^{**}P < 0.05$, $^{***}P < 0.01$.

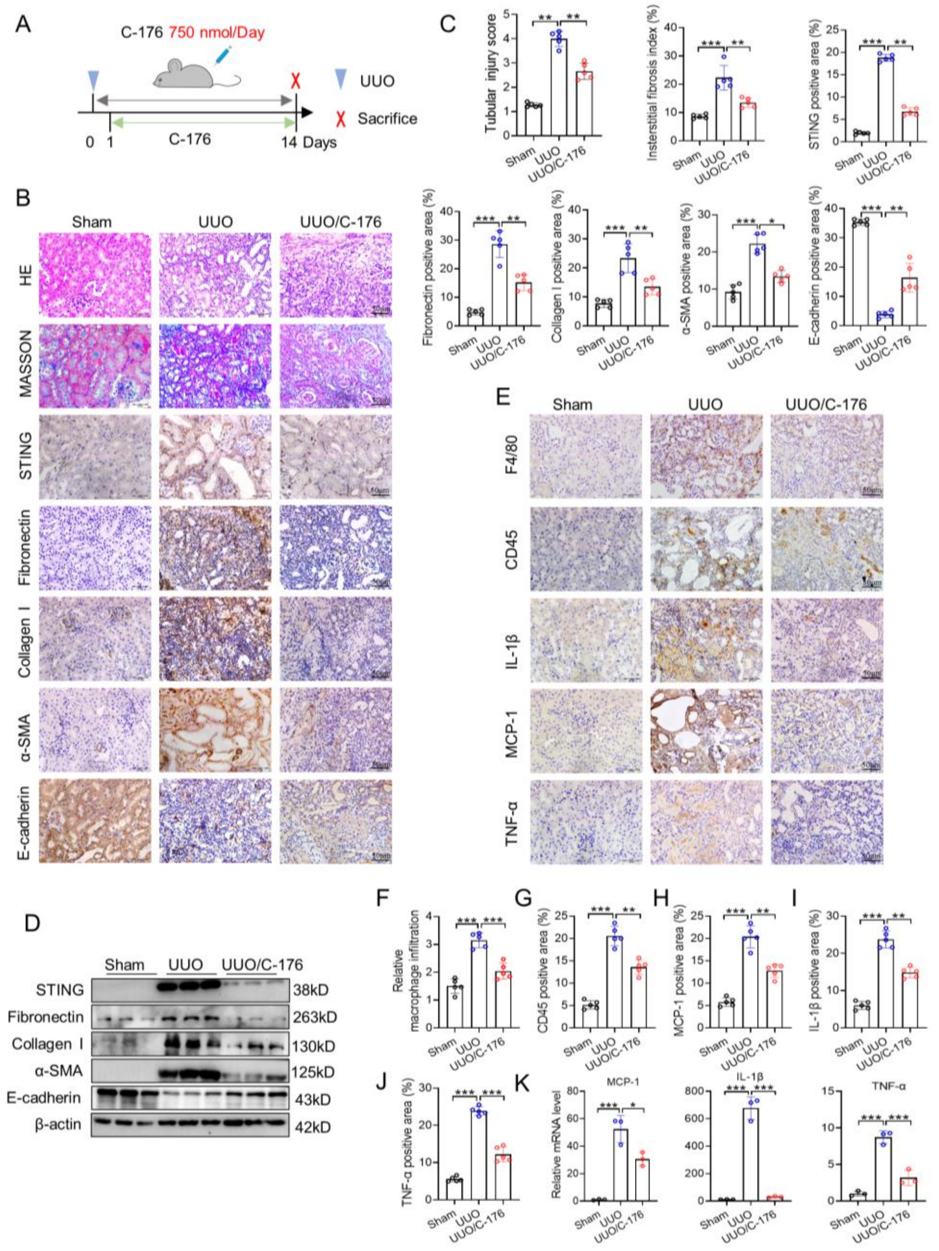

Fig S5 C-176 alleviates fibrosis and inflammation in UUO mice. The mice were randomly separated into the Sham, UUO and UUO+C-176 groups. **A** Experimental design: wild-type C57BL/6 mice were performed UUO surgery and treated with STING inhibitor (C-176) for 13 days. **B-C** Representative images of HE, Masson and IHC staining of fibronectin, collagen I, α-SMA, and E-cadherin and corresponding quantitative analyses in kidneys (n = 5). Bar = 50 μm. **D** Representative Western blot images of STING, fibronectin, collagen I, α-SMA, and E-cadherin in kidneys of the three groups (n = 5). **E-J** Representative images of IL-1β, MCP-1, TNF-α, CD45, and F4/80 and corresponding quantitative analyses in kidneys of the three mouse groups (n = 5). Bar = 50 μm. **K** Relative mRNA levels of MCP-1, IL-1β, and TNF-α in kidneys of the three mouse groups (n = 3). Data are presented as mean ± SEM. *$P < 0.05$, **$P < 0.01$, ***$P < 0.001$.

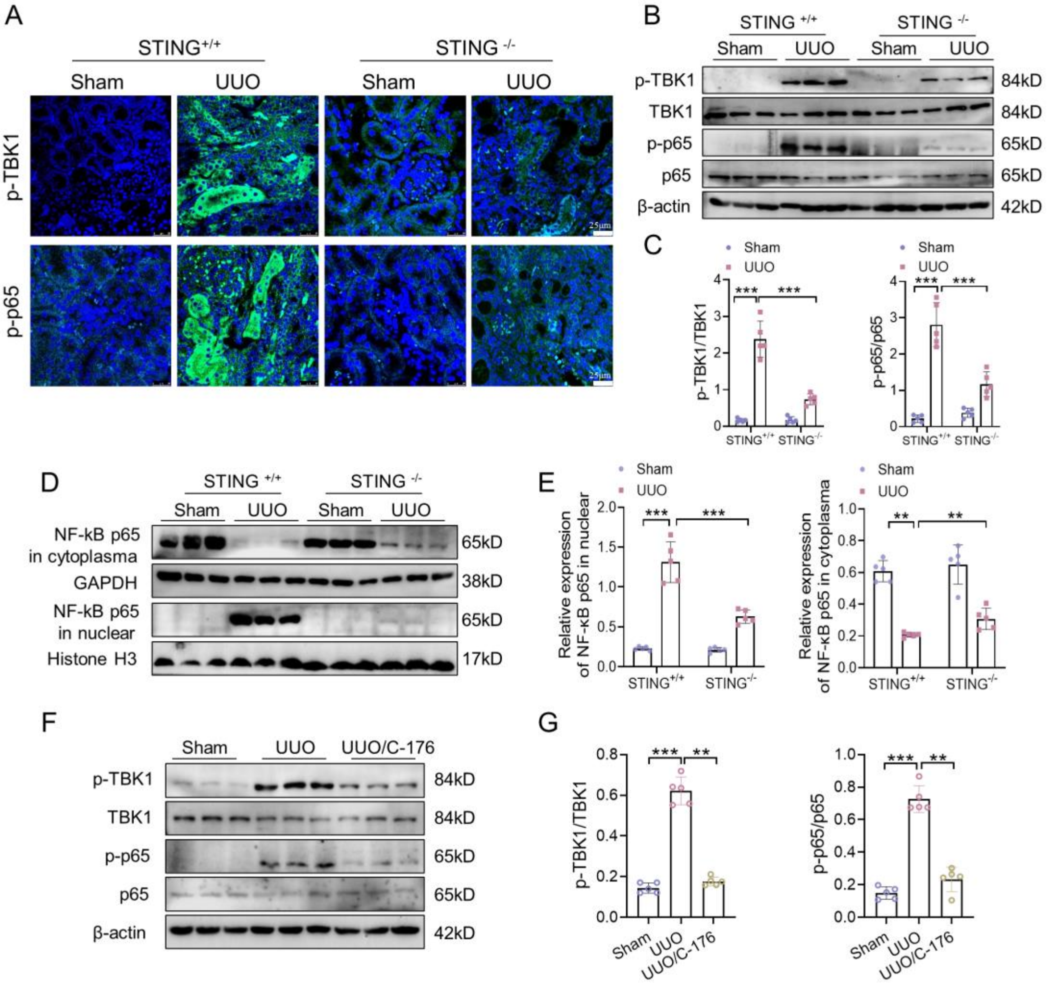

Fig. S6 Inhibition of STING suppresses UUO-induced activation of TBK1/NF-κB signaling. A Representative images of p-TBK1 and p-p65 expression in kidneys of four groups. Bar = 10 μm. **B-C** Representative western blot images of p-TBK1, TBK1, p-p65, and p65 and quantitation in kidneys of the four groups. **D, E** Representative western blot images of p65 in nuclear extracts and cytoplasmic extracts and quantitation in kidneys of the four groups (n=5) . **F, G** Representative western blot images of p-TBK1, TBK1, p-p65, and p65 and quantitation in kidneys of the three groups (n = 5). ***P* < 0.01, ****P* < 0.001.

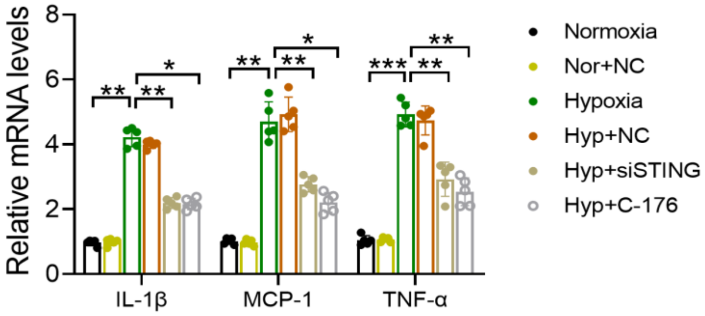

Fig. S7 Inhibition of STING inhibits hypoxia-induced inflammatory responses in HK-2 cells. Relative mRNA levels of IL-1β, MCP-1, and TNF-α in indicated groups. Data are presented as mean ± SEM. Nor: normoxia; NC: negative control; Hyp: hypoxia. n=5. *$P < 0.05$, **$P < 0.01$, ***$P < 0.001$.

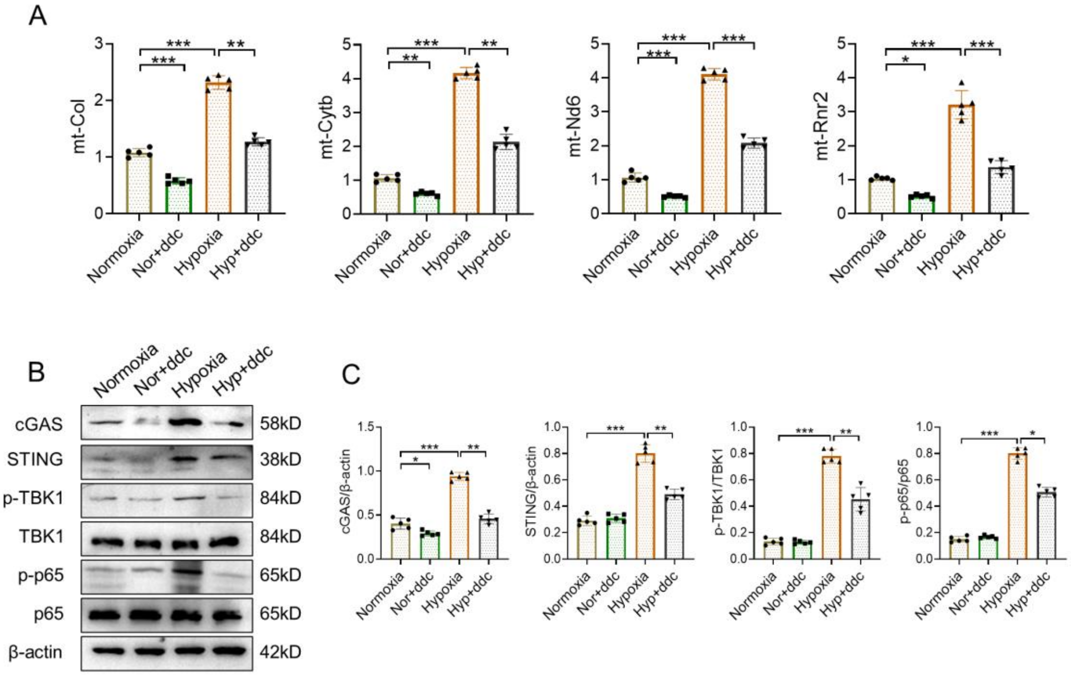

Fig. S8 Dideoxycytidine treatment suppressed the release of mtDNA and activation of cGAS-STING pathway in HK-2 cells exposed to hypoxia. A qPCR showing the effect of dideoxycytidine on the release of mtDNA (mt-Col, mt-Cytb, mt-Nd6, and mt-Rnr2) in HK-2 cells (n=5). **B, C** Representative Western blot images of STING, p-TBK1, TBK1, p-p65, and p65 and quantitation in HK-2 cells (n=5). Nor: normoxia; Hyp: hypoxia; ddC: dideoxycytidine. Data are presented as mean ± SEM. **P* < 0.05, ***P* < 0.01, ****P* < 0.001.

Table S1 Primary antibodies and manufacturers

Antibody	Company	Catalog No.	Application and Concentrations
PKC-δ	Abcam	ab182126	Western blot: 1:1000 IHC: 1:1000
α-SMA	Abcam	ab5694	Western blot: 1:1000 IHC: 1:1000
Kim-1	Abcam	ab47635	Western blot: 1:500 IHC: 1:100
IL-1β	Abcam	ab283818	IHC: 1:500
TNF-α	Abcam	ab1793	IHC: 1:100
P65	Abcam	ab16502	Western blot: 1:1000 IF: 1:100
VDAC1	Proteintech	55259-1-AP	Western blot: 1:500 IHC: 1:100
VDAC1	Proteintech	66345-1-Ig	Western blot: 1:500 IF: 100
cGAS	Proteintech	26416-1-AP	Western blot: 1:1000
STING	Proteintech	19851-1-AP	Western blot: 1:2000 IHC: 1:2000
TBK1	Proteintech	28397-1-AP	Western blot: 1:1000
Collagen I	Proteintech	14695-1-AP	Western blot: 1:1000 IHC: 1:500
Fibroenctin	Proteintech	15613-1-AP	Western blot: 1:1000
Fibronectin	Santa Cruz Biot echnology	sc-8422	IHC: 1:500
E-cadherin	Proteintech	20874-1-AP	Western blot: 1:1000 IHC: 1:3000
AQP1	Proteintech	20333-1-AP	IF: 1:100
Calbindin-D28k	Proteintech	66394-1-Ig	IF: 1:100
MCP-1	Proteintech	26161-1-AP	IHC: 1:500
F4/80	Proteintech	29414-1-AP	IHC: 1:500
CD45	Proteintech	60287-1-Ig	IHC: 1:5000
p-P65	Cell Signaling Technology	#3033	Western blot: 1:1000 IF: 1:100
p-TBK1	Cell Signaling Technology	#5483	Western blot: 1:1000 IF: 1:100
p-PKC-δ	Cell Signaling Technology	#14787	Western blot: 1:1000

Table S2 Chemical reagents

Name	Supplier
Rottlerin	MedChemExpress, HY-18980
C-176	MedChemExpress, HY-112906
VBIT-12	Aladdin, 2089227-65-4
JC-1	Beyotime, C2006
MitoTracker	Thermo Fisher Scientific, M7512
MitoSox Red	Thermo Fisher Scientific, M36008
Sirius Red/Fast Green Collagen Staining Kit	Chondrex, #9046
EGS	MedChemExpress, HY-130458
dideoxycytidine	MedChemExpress, HY-17392

Table S3 Prime sequence of genes

Gene name	Species	Sequence 5’-3’	
GAPDH	human	Forward	CTGACTTCAACAGCGACACC
		Reverse	TGCTGTAGCCAAATTCGTTGT
MCP-1	human	Forward	ACGCTTCTGGGCCTGTTGTTCA
		Reverse	TGGGGCATTAACTGCATCTGGCT
TNF-α	human	Forward	GGCAGTCAGATCATCTTCTCGA
		Reverse	CGGTTCAGCCACTGGAGCT
IL-6	human	Forward	AGTTGCCTTCTTGGGACTGA
		Reverse	TCCACGATTTCCCAGAGAAC
IL-1β	human	Forward	TCAATGGCAATGAGGATG
		Reverse	TGTAGTGGTGGTCGGAGA
E-cadherin	human	Forward	ATGCTGATGCCCCCAATACC
		Reverse	ATCTTGCCAGGTCCTTTGCT
α-SMA	human	Forward	CTTGTTTGGGAAGCAAGTGGG
		Reverse	GATTCCTGACAGTGCTTGGC
GAPDH	mouse	Forward	CGGAGTCAACGGATTTGGTCGTAT
		Reverse	AGCCTTCTCCATGGTGGTGAAGAC

MCP-1	mouse	Forward	TTGACCCGTAAATCTGAAGCTAAT
		Reverse	TCACAGTCCGAGTCACACTAGTTCAC
TNF-α	mouse	Forward	CCCTCACACTCAGATCATCTTCT
		Reverse	GCTACGACGTGGGCTACAG
IL-6	mouse	Forward	AGTTGCCTTCTTGGGACTGA
		Reverse	TCCACGATTTCCCAGAGAAC
IL-1β	mouse	Forward	ACCTTCCAGGATGAGGACATGA
		Reverse	AACGTCACACACCAGCAGGTTA
mt-CoI	human	Forward	GCCCCAGATATAGCATTCCC
		Reverse	GTTCATCCTGTTCCTGCTCC
mt-Rnr2	human	Forward	GTTACCCTAGGGATAACAGCGC
		Reverse	GATCCAACATCGAGGTCGTAAACC
mt-Nd6	human	Forward	TTAGCATTAAAGCCTTCACC
		Reverse	CCAACAAACCCACTAACAAT
mt-Cytb	human	Forward	AGTAGACAAAGCCACCTTGA
		Reverse	CCGCGATAATAAATGGTAAG
18SrDNA	human	Forward	TAGAGGGACAAGTGGCGTTC
		Reverse	CGCTGAGCCAGTCAGTGT
