# Supplementary material for: Inhibition of PKC-δ retards kidney fibrosis via inhibiting cGAS-STING signaling pathway in mice
Source: Cell Death Discov. 2024 Jul 7;10:314. doi: 10.1038/s41420-024-02087-z (PMC11228024; doi:10.1038/s41420-024-02087-z)

Full unedited gels for Figure 1

Fig. 1A

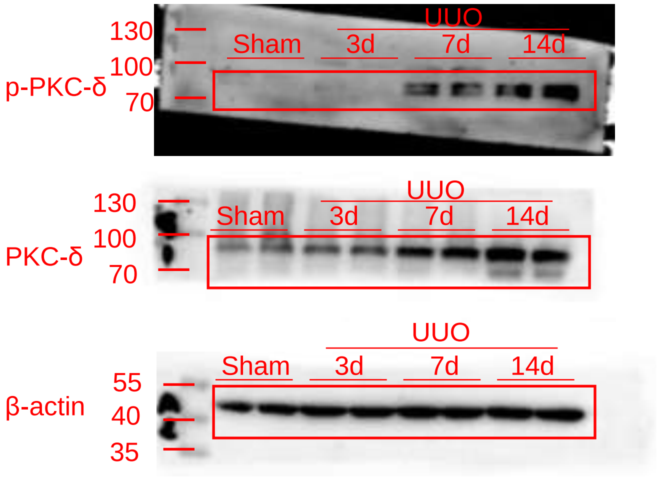

Fig. 1C

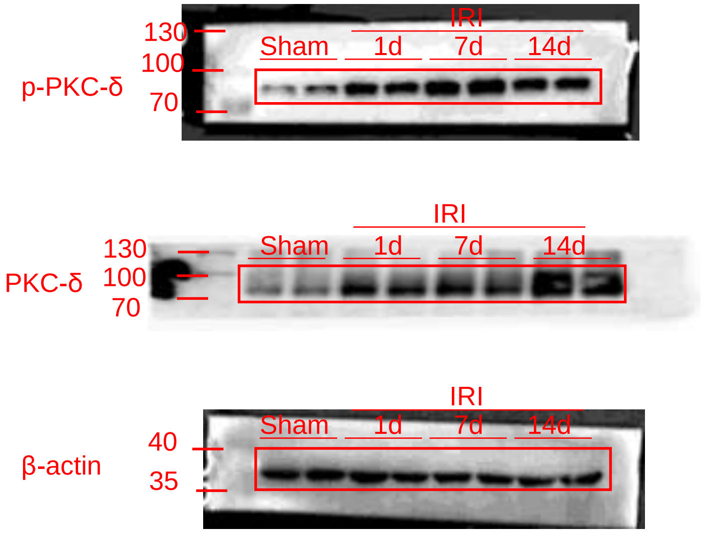

Full unedited gels for Figure 2

Fig. 2E

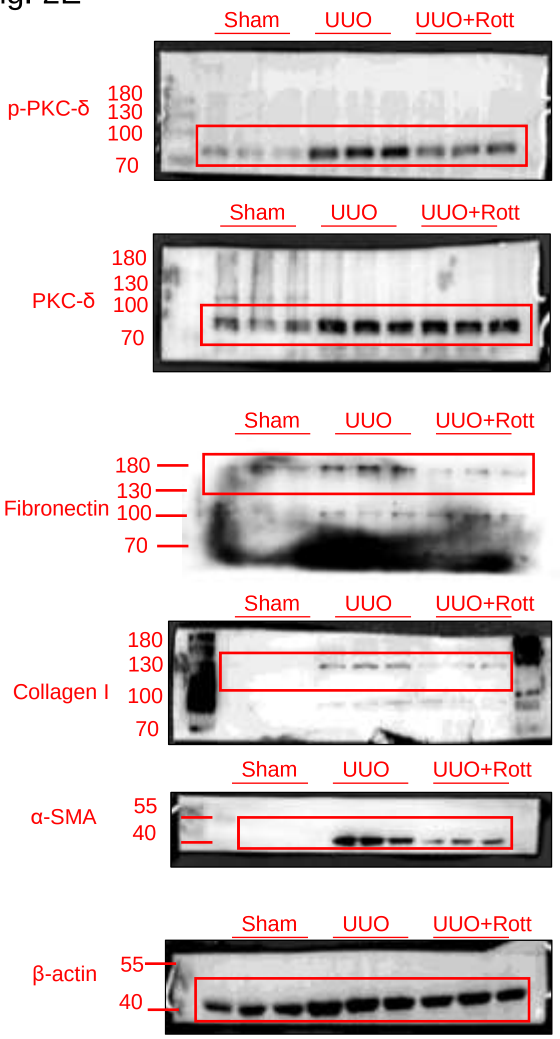

Fig. 2K

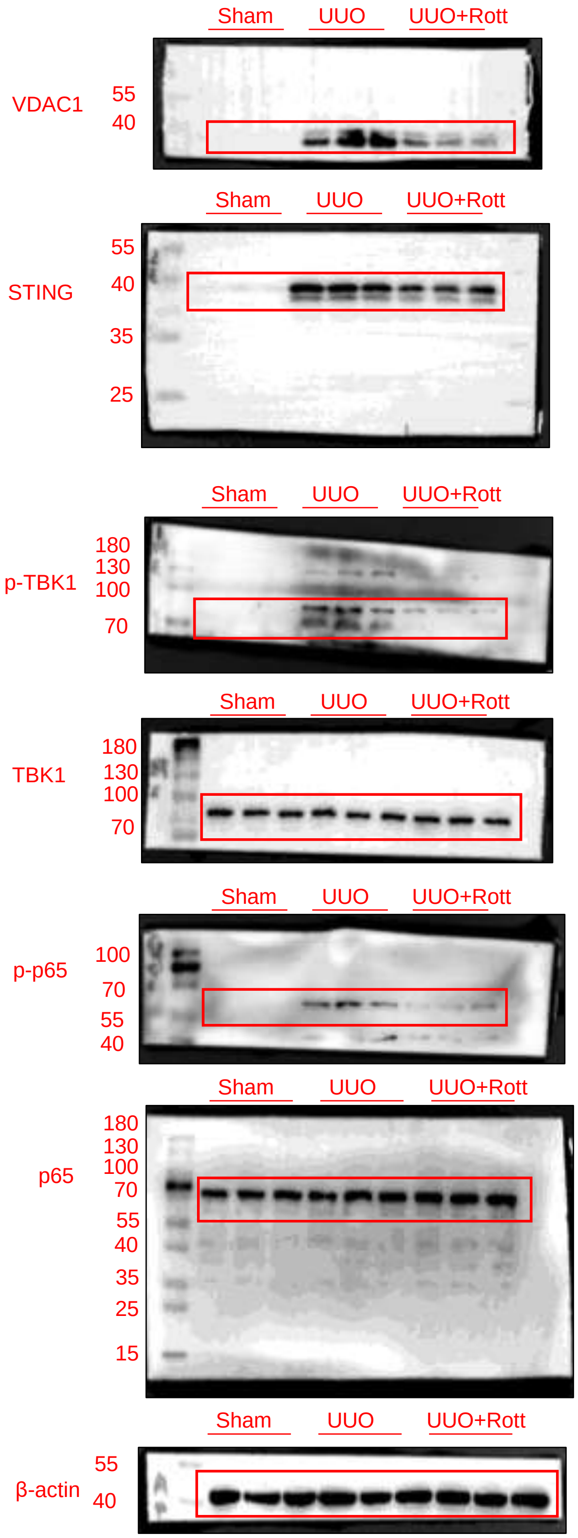

Full unedited gels for Figure 3

Fig. 3I

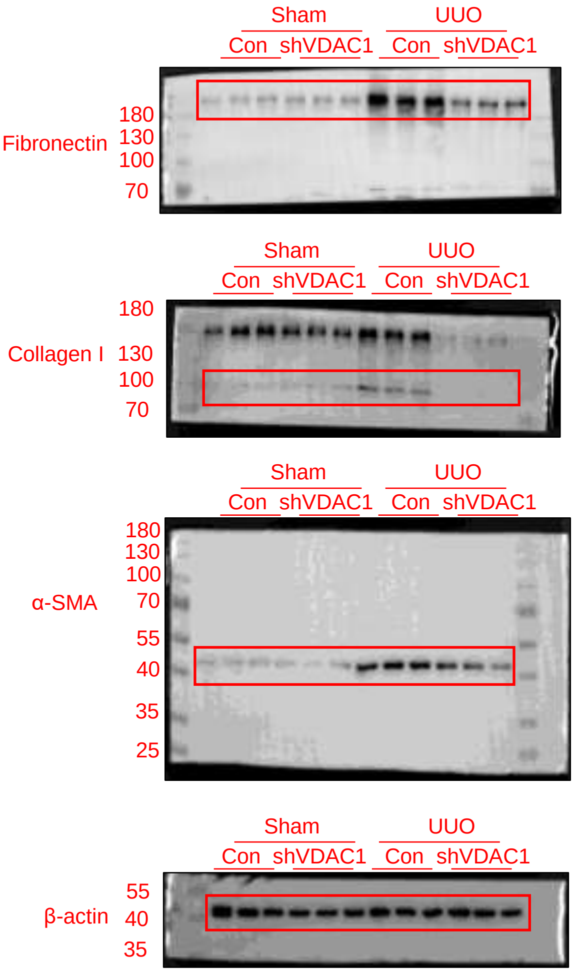

Full unedited gels for Figure 4

Fig. 4A

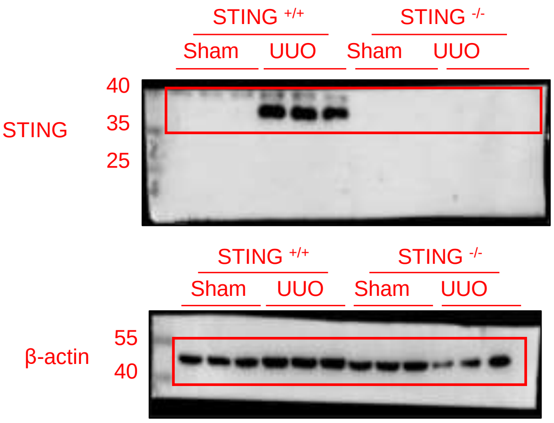

Fig. 4D

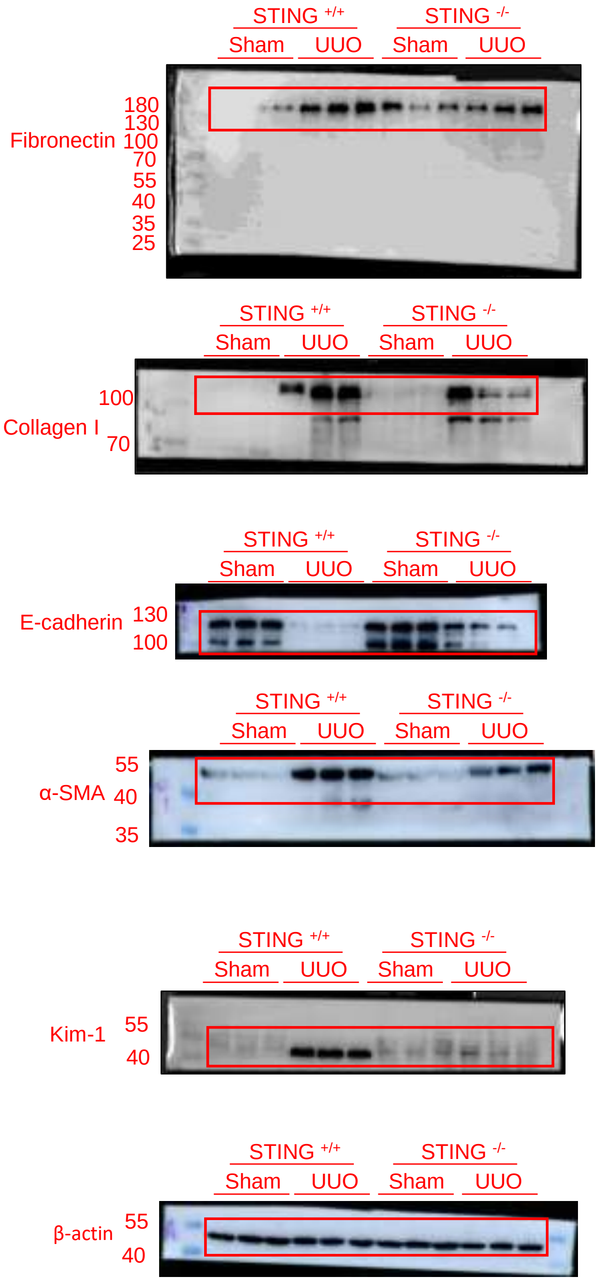

Full unedited gels for Figure 5

Fig. 5A

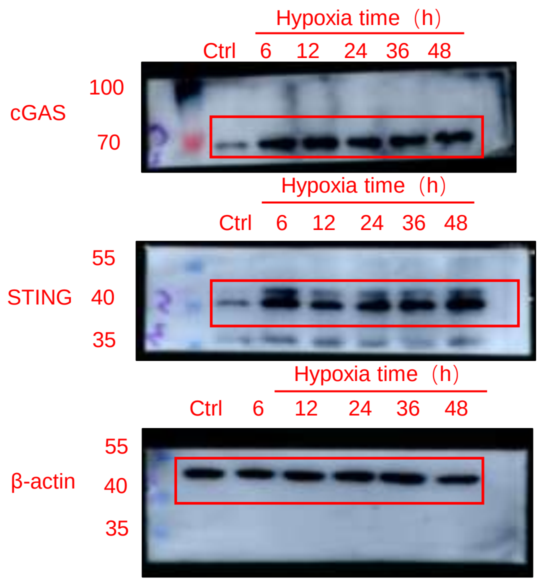

Fig. 5D

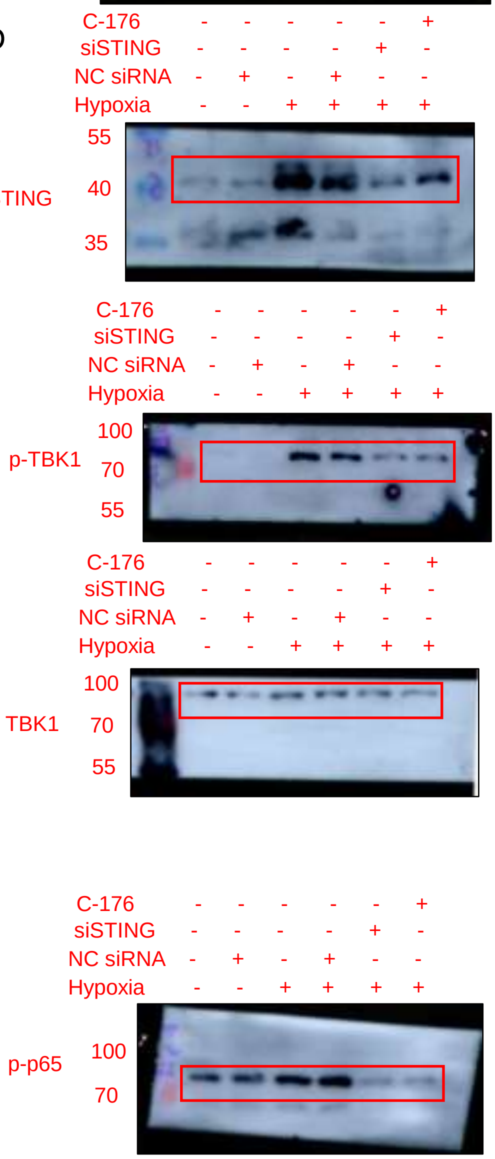

Fig. 5D

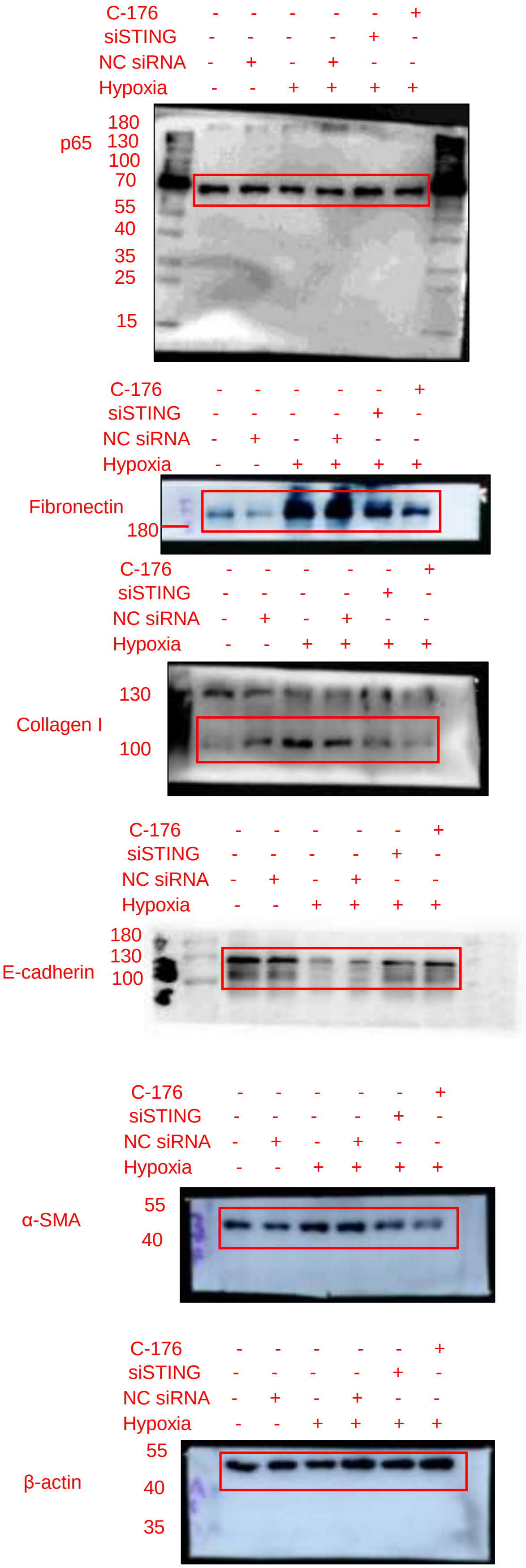

Full unedited gels for Figure 6

Fig. 6A

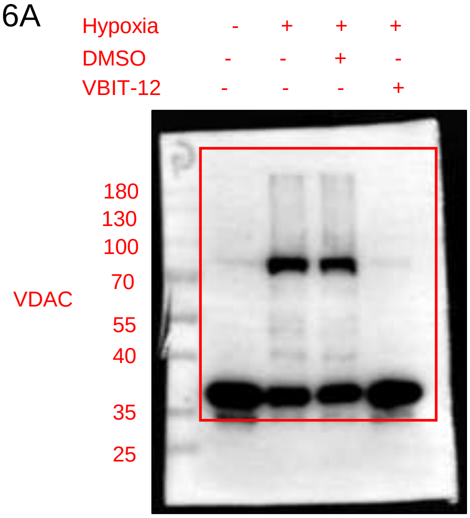

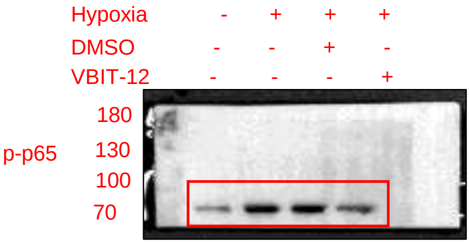

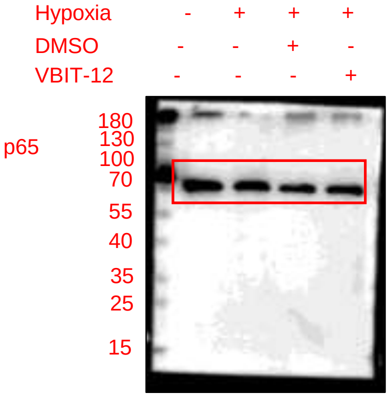

Fig. 6B

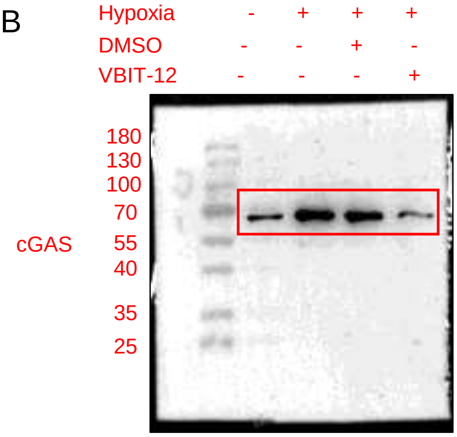

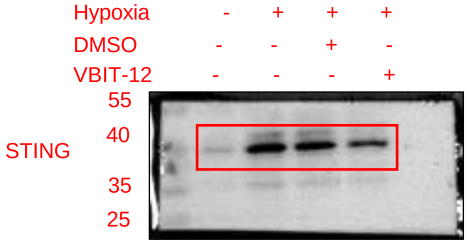

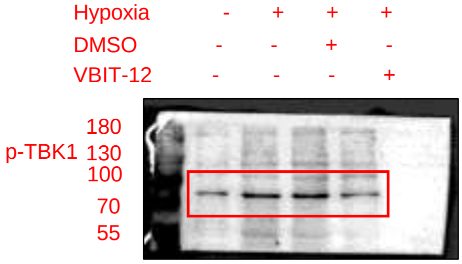

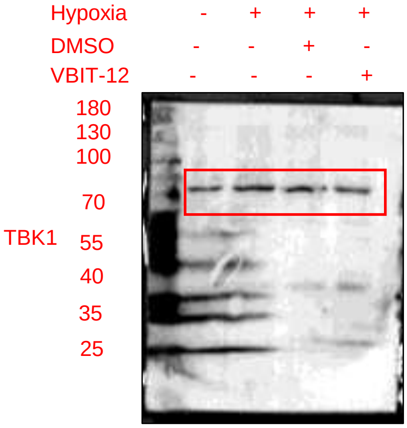

Full unedited gels for Figure 6

Fig. 6B

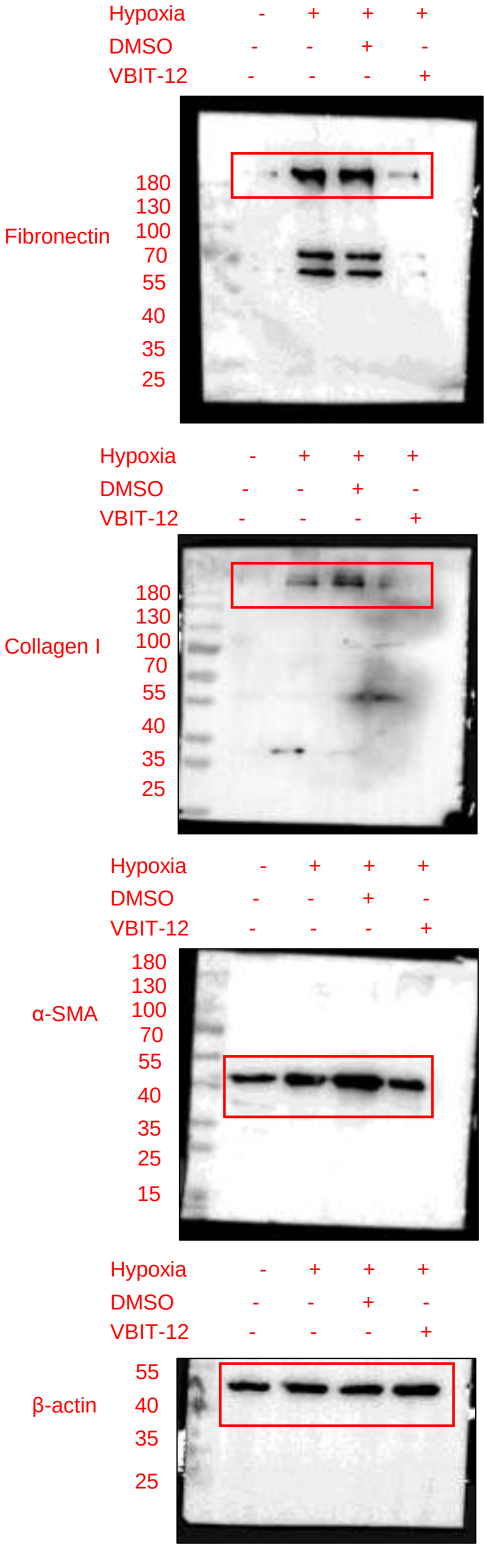

Full unedited gels for Figure 7

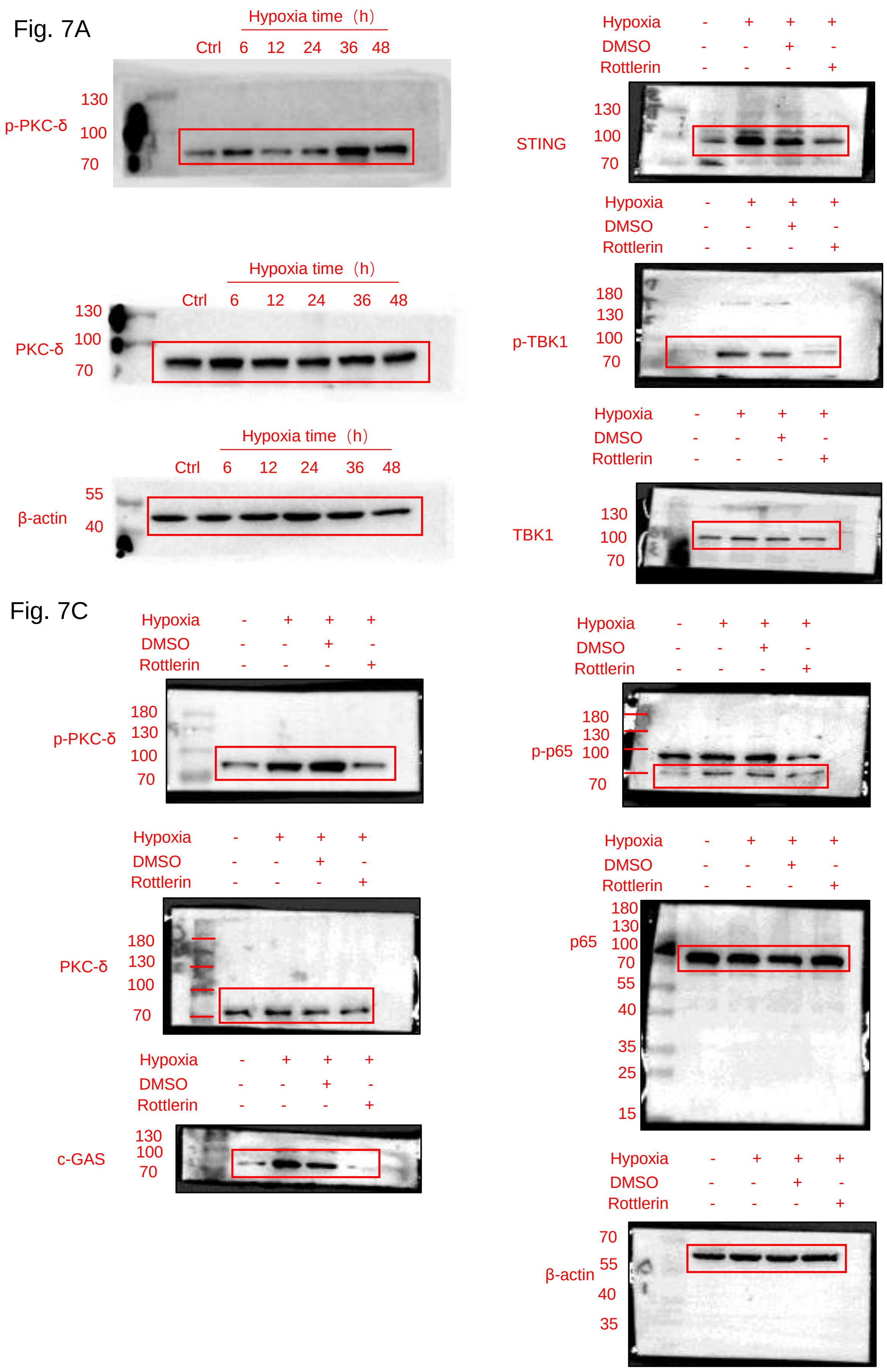

Full unedited gels for Figure 7

Fig. 7J

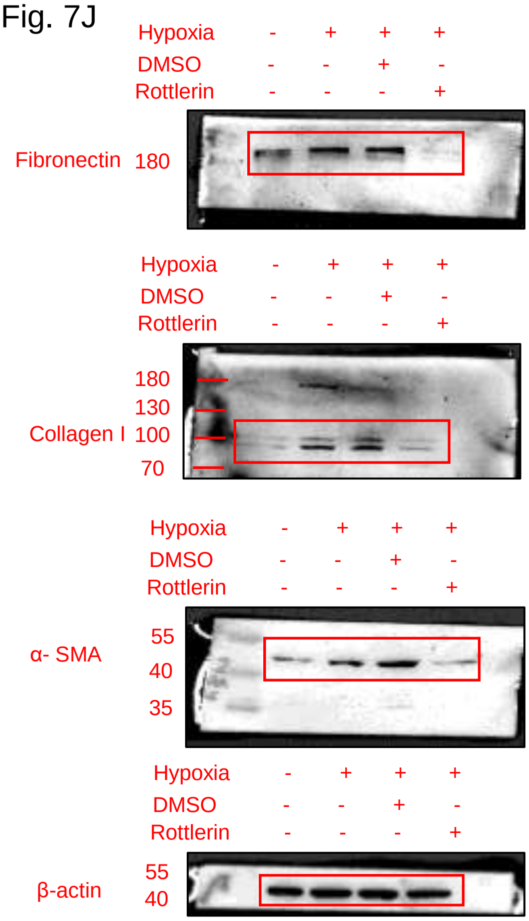

Full unedited gels for Figure 8

Fig. 8H

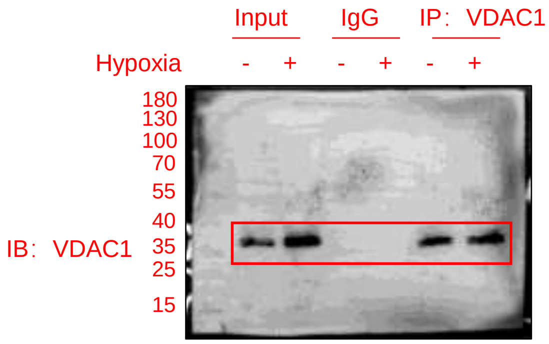

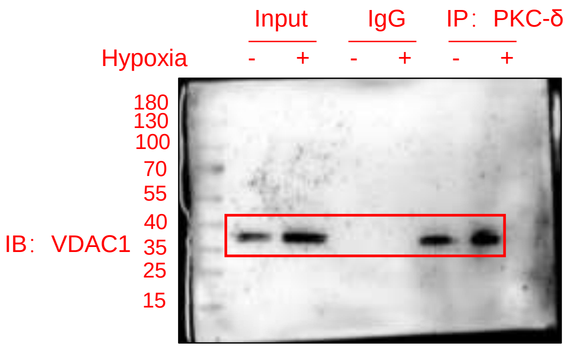

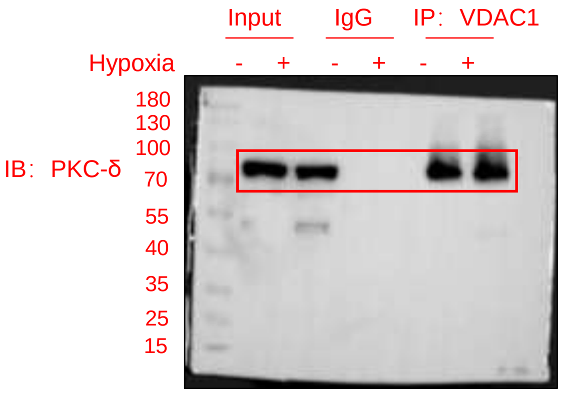

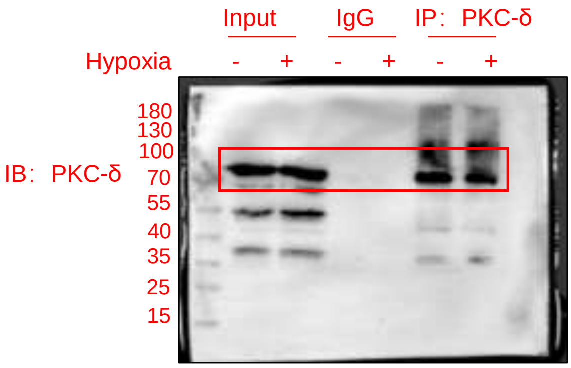

Fig. 8I

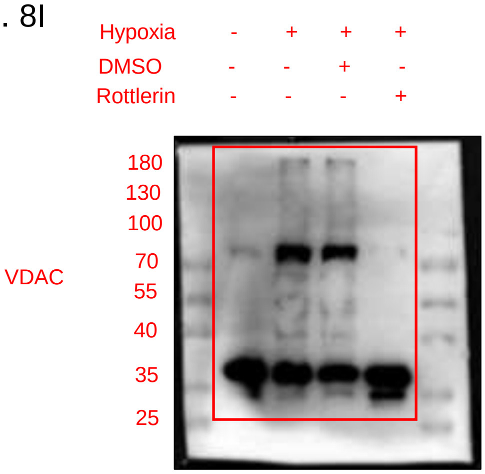

Full unedited gels for Supplementary Figure S2

Fig. S2C

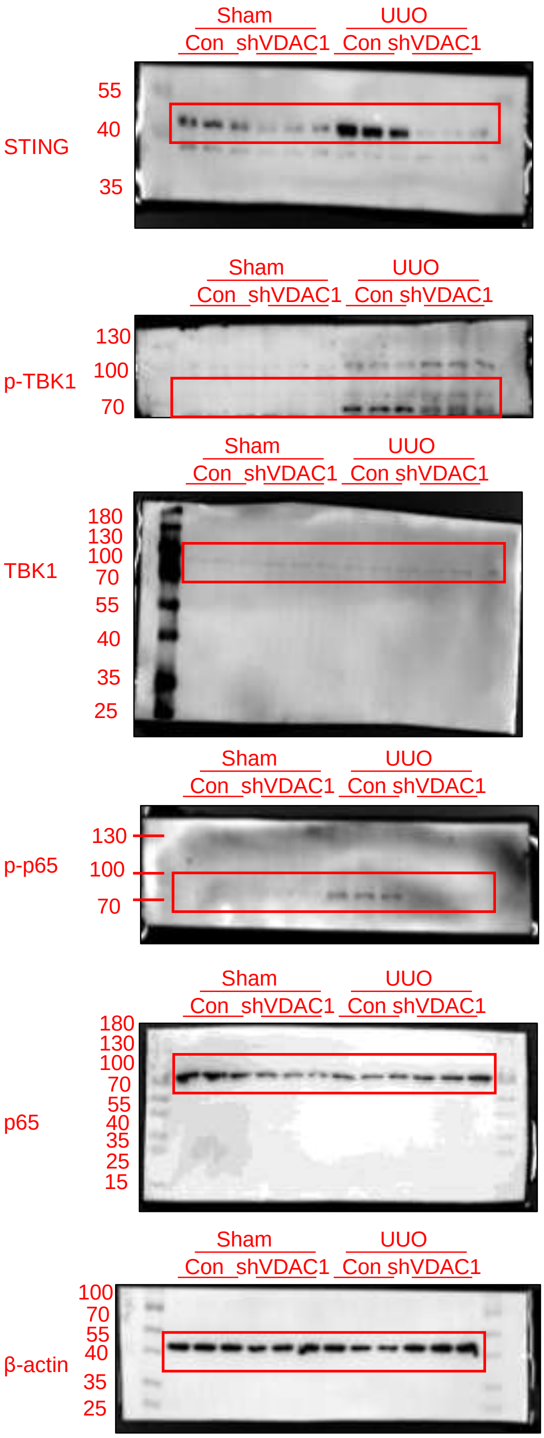

Full unedited gels for Supplementary Figure S3

Fig. S3D

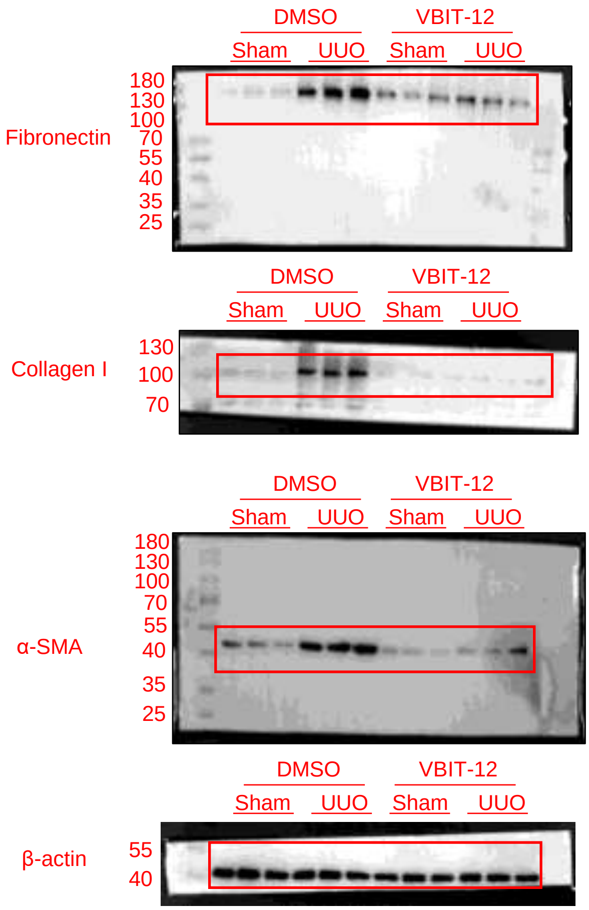

Full unedited gels for Supplementary Figure S4

Fig. S4C

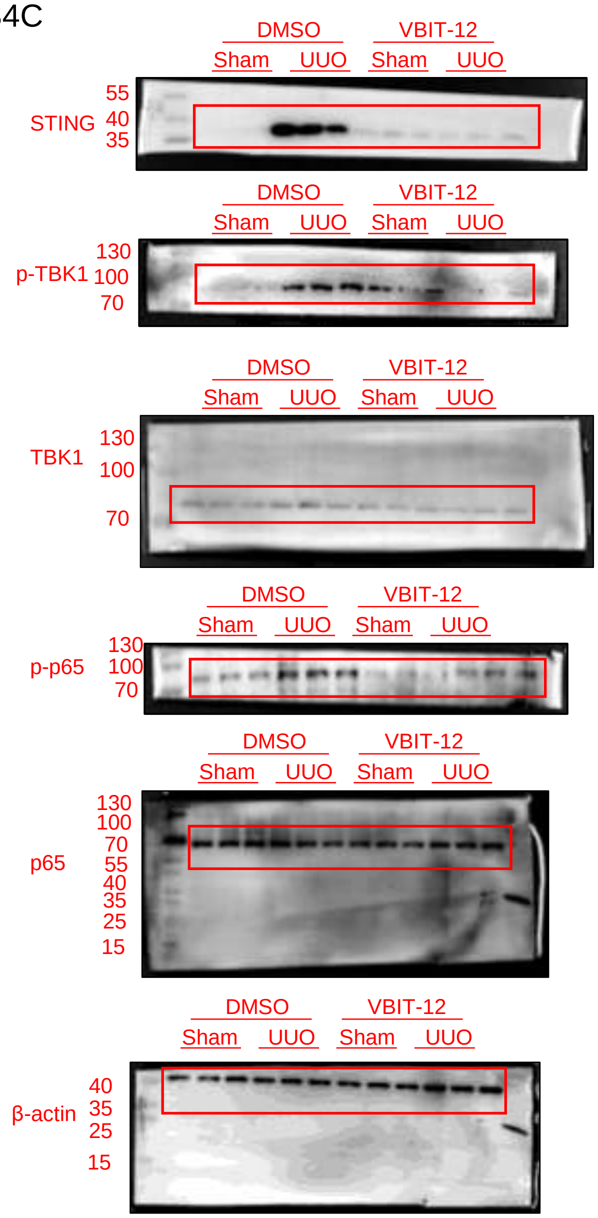

Full unedited gels for Supplementary Figure S5

Fig. S5D

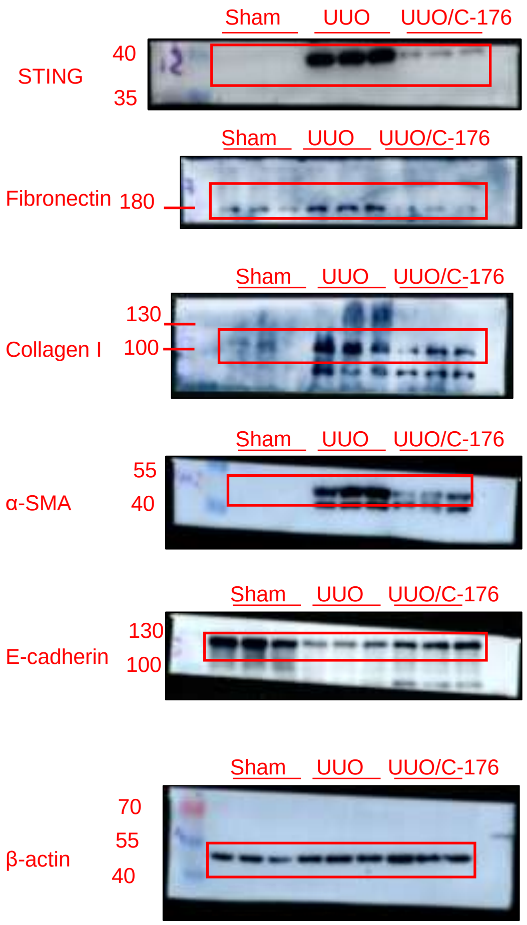

Full unedited gels for Supplementary Figure S6

Fig. S6B

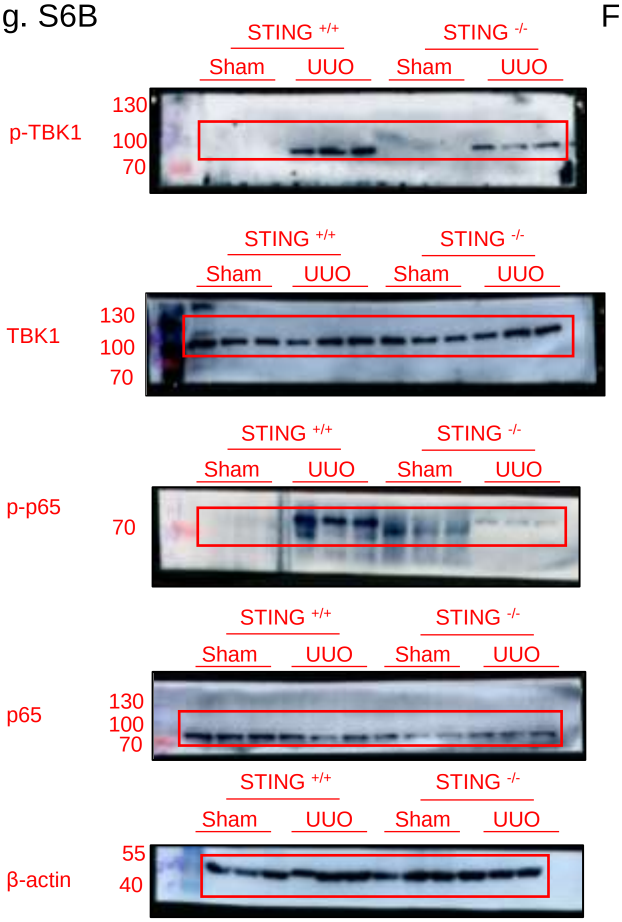

Fig. S6D

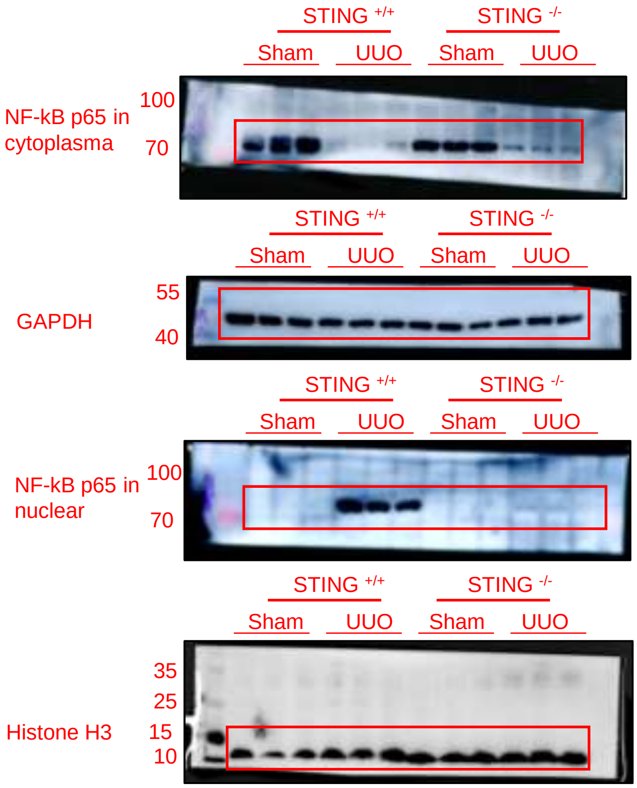

Fig. S6F

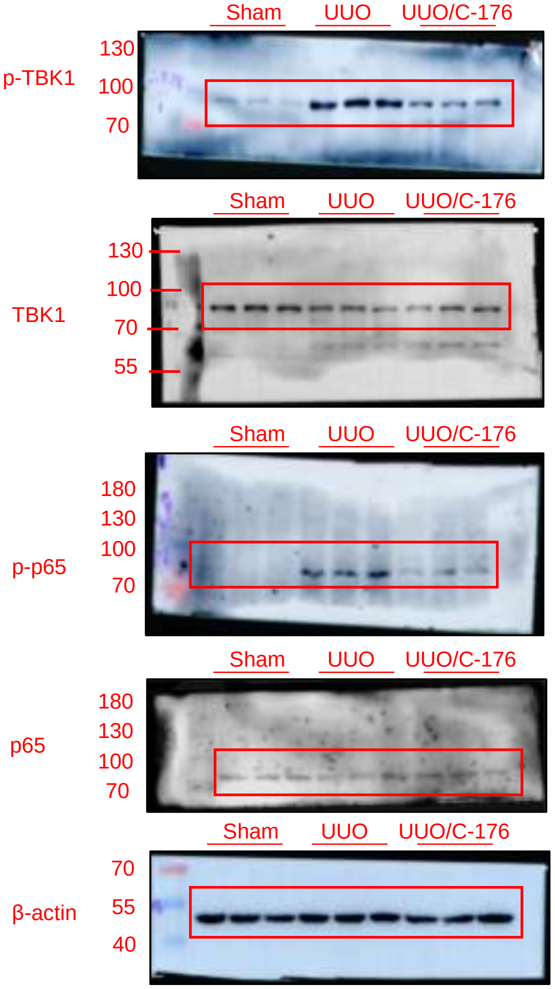

Full unedited gels for Supplementary Figure S8

Fig. S8B

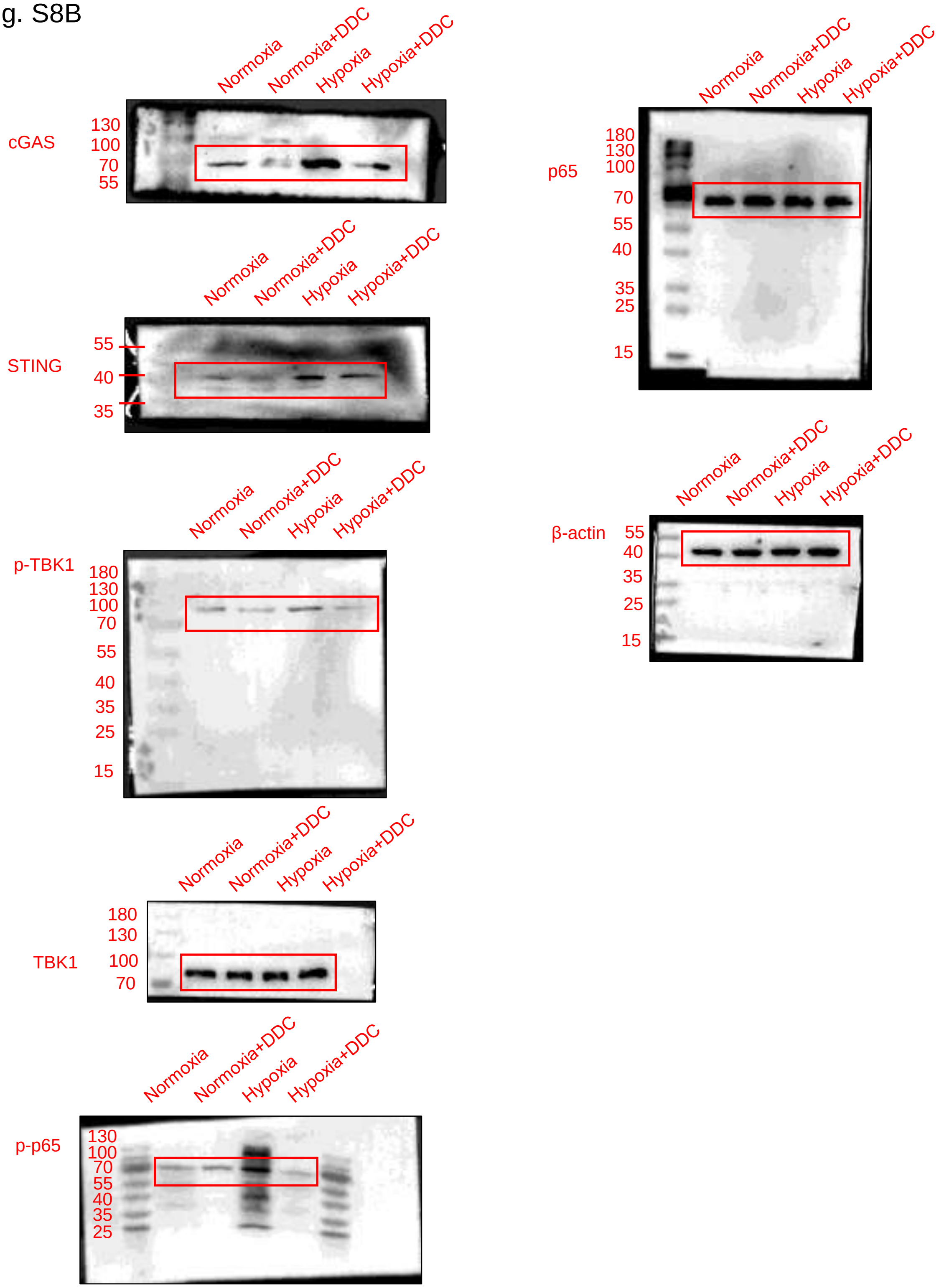

Supplement: Supplementary file 2 — Original western blots [file 41420_2024_2087_MOESM2_ESM.pdf]
